# TriFusion-PCGNet: A Hybrid Multi-Branch Deep Learning Network for Energy-Aware Normal/Abnormal Heart Sound Classification

**DOI:** 10.3390/life16091417

**Published:** 2026-08-26

**Authors:** Tolga Çakmak, Çetin Mirzaoğlu, Melike Demir, Fatih Demir

**Affiliations:** 1 Department of Cardiology, Balıkesir Atatürk City Hospital, University of Health Sciences Turkiye, Balıkesir 10050, Turkey; tolgacakmak85@gmail.com; 2 Department of Cardiology, Elazig Fethi Sekin City Hospital, University of Health Sciences, Elazig 23280, Turkey; dr.cmirzaoglu@gmail.com; 3 Eco-Informatics Department, Fırat University, Elazig 23119, Turkey; ironfmd23@gmail.com; 4 INCISOFT Software Information Technologies, Fırat Teknokent, Fırat University, Elazig 23000, Turkey

**Keywords:** phonocardiogram (PCG), heart sound classification, normal/abnormal classification, multi-branch deep learning, TriFusion-PCGNet

## Abstract

**Background/Objectives:** Cardiovascular diseases remain a leading cause of death worldwide, and early diagnosis is essential. Phonocardiogram (PCG) signals provide a non-invasive and inexpensive means of cardiac assessment. However, accurate interpretation of PCG signals requires clinical expertise. In this paper, we propose TriFusion-PCGNet, a hybrid multi-branch deep learning framework for automated heart sound classification. **Methods:** The proposed method was evaluated on the PhysioNet/Computing in Cardiology Challenge 2016 dataset. Three complementary PCG representations—the raw signal, band-pass filtered signal, and Hilbert envelope signal—were processed through dedicated feature extraction branches using residual convolutional networks, dilated residual convolutions, and a BiGRU with a temporal attention mechanism. The extracted features were fused and classified using fully connected layers. Performance was evaluated using the original train–validation split and stratified 10-fold cross-validation. **Results:** Under a strictly separated record-level 10-fold cross-validation protocol, TriFusion-PCGNet achieved 90.87% mean accuracy and 95.34% AUC, with outer test folds isolated from training and model selection. Subject-level analysis showed substantially lower performance, emphasizing the importance of subject-disjoint evaluation for patient-level generalization. **Conclusions:** TriFusion-PCGNet integrates complementary PCG representations for automated normal/abnormal heart sound classification. However, the substantially lower subject-disjoint performance indicates that the record-level results should not be interpreted as evidence of patient-independent generalization, and further validation on independent subject-annotated datasets is required.

## 1. Introduction

Cardiovascular diseases remain a major cause of mortality worldwide and continue to impose a substantial burden on healthcare systems [1]. Cardiac sounds, generated by mechanical events associated with valve activity and blood flow, can be recorded as phonocardiograms (PCGs) and provide useful information for assessing cardiac function [2]. Auscultation remains an economical and readily accessible method for the initial assessment of cardiac abnormalities [3]. However, its interpretation is influenced by the examiner’s experience, auditory perception, recording quality, and environmental noise, potentially affecting the identification of abnormal heart sounds [4]. Consequently, computer-aided analysis and artificial intelligence (AI)-based methods have received increasing attention for automated and objective analysis of PCG recordings [5,6].

PCG signals can be acquired using conventional acoustic stethoscopes coupled to external recording systems, electronic stethoscopes, or dedicated digital auscultation devices. Modern electronic and digital stethoscopes may incorporate microphones or piezoelectric sensors, analog amplification, adjustable frequency-response modes, onboard filtering, noise reduction, and digital data transmission [7]. These instrumental differences can substantially influence the recorded PCG waveform, since the signal amplitude and spectral content can be modified by the characteristics of the sensor, the frequency response, the sampling configuration, the amplification, the contact pressure, and the ambient noise. Therefore, PCG recordings obtained from different devices can have different bandwidths, signal-to-noise ratios, and artifact characteristics. The variability in acquisition is a major challenge for the automatic analysis of heart sounds and motivates the development of preprocessing techniques that reduce the frequency components that are not relevant but retain the information related to the cardiac sounds [8].

Recently, machine learning methodologies—especially deep learning—have profoundly transformed PCG classification. Traditional techniques generally depend on manually produced acoustic characteristics paired with classical classifiers [9,10], while deep learning architectures acquire hierarchical representations directly from waveform-level inputs or their time-frequency transformations. In this framework, convolutional models have been extensively utilized for time-frequency representations [11,12]. For example, Xiao et al. [11] created streamlined 1D CNN architectures for effective classification, whereas Alkhodari and Fraiwan [13] integrated CNN and BiLSTM frameworks to seize temporal dynamics in PCG data. In a comparable manner, Tian et al. [14] tackled class imbalance by the application of Res2Net with Seesaw loss, while Duan et al. [15] introduced CNN models based on tensor decomposition to mitigate parameter redundancy. Safak et al. [16] have documented an accuracy of 98% by utilizing spectrogram-based representations in conjunction with RAMM and NRBMI feature selection. Hybrid and ensemble methodologies have also attracted interest. Kalatehjari et al. [17] combined CNN and BiLSTM for the analysis of multi-input PCG–ECG, while Safdar et al. [18] utilized transformer models alongside squeeze-and-excitation blocks to capture long-range relationships. Similarly, Ma et al. [19] proposed a parameter-efficient densely connected dual-attention network for PCG classification, demonstrating the potential of attention-based contextual feature modeling while explicitly considering model complexity. Beyond supervised PCG classification, Staffini et al. [20] combined disentangled latent representation learning with bidirectional temporal modeling for unsupervised anomaly detection in wearable heart-rate sequences. Although addressing a different cardiovascular signal and task, their approach highlights the potential of temporal representation learning and unsupervised modeling for future analysis of limited-label physiological data. Furthermore, Wang et al. [21] introduced LeM-MARNetwork for elucidative multi-branch feature extraction, while Chandrasekhar et al. [22] presented a PJM-DJRNN architecture emphasizing sophisticated pre-processing. Notwithstanding the advancements in deep learning, conventional methods like XGBoost paired with crafted acoustic characteristics can still deliver competitive results [23]. Nonetheless, numerous constraints persist. First, many studies rely on a single signal representation, although PCG signals are intrinsically non-stationary and contain complex transient components that may not be fully captured in a single representation domain [24]. Spectrogram inputs are constrained by fixed-resolution, whereas CWT offers multi-resolution functionality and superior representation of temporal features [25]. Secondly, current designs are predominantly single-stream, concentrating on either CNN models for features or transformer networks for general context, failing to leverage their complementary advantages. As a result, the integration of local acoustic patterns with long-range relationships is still constrained. Third, the majority of models include a Softmax layer for ultimate classification; yet the presumption of distinctly separated feature spaces may result in less than optimal choices in overlapping or non-linearly separable distributions. In these instances, geometry-informed classifiers might offer enhanced differentiation by utilizing local neighborhood configurations [26,27,28]. Bilen et al. [29] proposed the DTF-STCANetwork to tackle these deficiencies. ConvNeXt captures local acoustic patterns, whereas the Swin Transformer models broader contextual dependencies through hierarchical attention. Furthermore, an attention refinement module is integrated to improve the representation of discriminative features. This study proposes TriFusion-PCGNet, a hybrid multi-branch deep learning architecture for the automated classification of heart sounds. Unlike conventional approaches that rely on a single representation of PCG signals, the proposed framework simultaneously utilizes three complementary deterministic representations of the same PCG signal: the raw phonocardiogram waveform, the band-pass filtered signal, and the Hilbert envelope signal. Each representation is processed through a dedicated feature-extraction branch designed to emphasize different characteristics of the same underlying PCG signal. Residual convolutional networks extract waveform-level morphological patterns from the raw signal, dilated residual convolutions model broader temporal patterns in the filtered signal, and a Conv1D–BiGRU–temporal attention branch captures sequential amplitude-envelope dynamics. The high-level features extracted from these three branches are fused into a unified representation before binary classification. By integrating complementary morphological, temporal, and energy-based information, the proposed architecture aims to improve the robustness and discriminative capability of automatic PCG classification while maintaining an end-to-end learning framework. The model is evaluated using the PhysioNet/Computing in Cardiology Challenge 2016 dataset [2,30], a widely used benchmark for normal/abnormal heart sound classification. The experimental evaluation includes strictly separated record-level cross-validation, an additional subject-level sensitivity analysis on the subset with available subject identifiers, repeated random initializations, calibration assessment, signal-duration sensitivity analysis, extended ablation experiments, and computational-efficiency analysis. The results demonstrate the capability of TriFusion-PCGNet for automated normal/abnormal PCG classification. More clinically detailed resources are also available, such as the CirCor DigiScope dataset introduced by Oliveira et al. [31], which contains subject-annotated PCG recordings acquired at multiple auscultation locations and provides murmur-related labels while maintaining subject-level separation across data partitions. Such resources represent an important basis for future external validation and evaluation of patient-level generalization. Nevertheless, the present task is limited to binary recording-level classification, and the model has not yet been externally or prospectively validated across independent populations, recording devices, clinical sites, age groups, or specific cardiac pathologies. Therefore, further external validation is required before considering clinical application.

## 2. Dataset

The PhysioNet/Computing in Cardiology Challenge 2016 dataset was developed to support automated analysis of phonocardiogram (PCG) recordings and the classification of heart sounds as normal or abnormal [2]. The recordings originate from heterogeneous acquisition conditions and contain considerable variability in recording duration, signal morphology, and signal quality. In the dataset used in this study, a total of 3240 PCG recordings in WAV format were included in the experimental analysis. Each recording was associated with a binary normal/abnormal label, and this binary classification task constitutes the prediction target of the present study. Accordingly, the model is designed to distinguish normal from abnormal PCG recordings rather than to identify a specific cardiovascular disease or valvular pathology.

PCG recordings represent acoustic manifestations of mechanical cardiac activity, including events associated with valve motion and blood-flow dynamics. However, the morphology of these signals can vary substantially across recordings because of physiological variability as well as differences in acquisition conditions, sensor characteristics, body location, ambient noise, motion, and other recording artifacts. Consequently, normal and abnormal recordings cannot necessarily be distinguished reliably through visual inspection of the raw waveform alone. Signal contamination can further obscure the temporal and morphological characteristics associated with cardiac sounds, creating an additional challenge for automated analysis.

**Figure 1:**
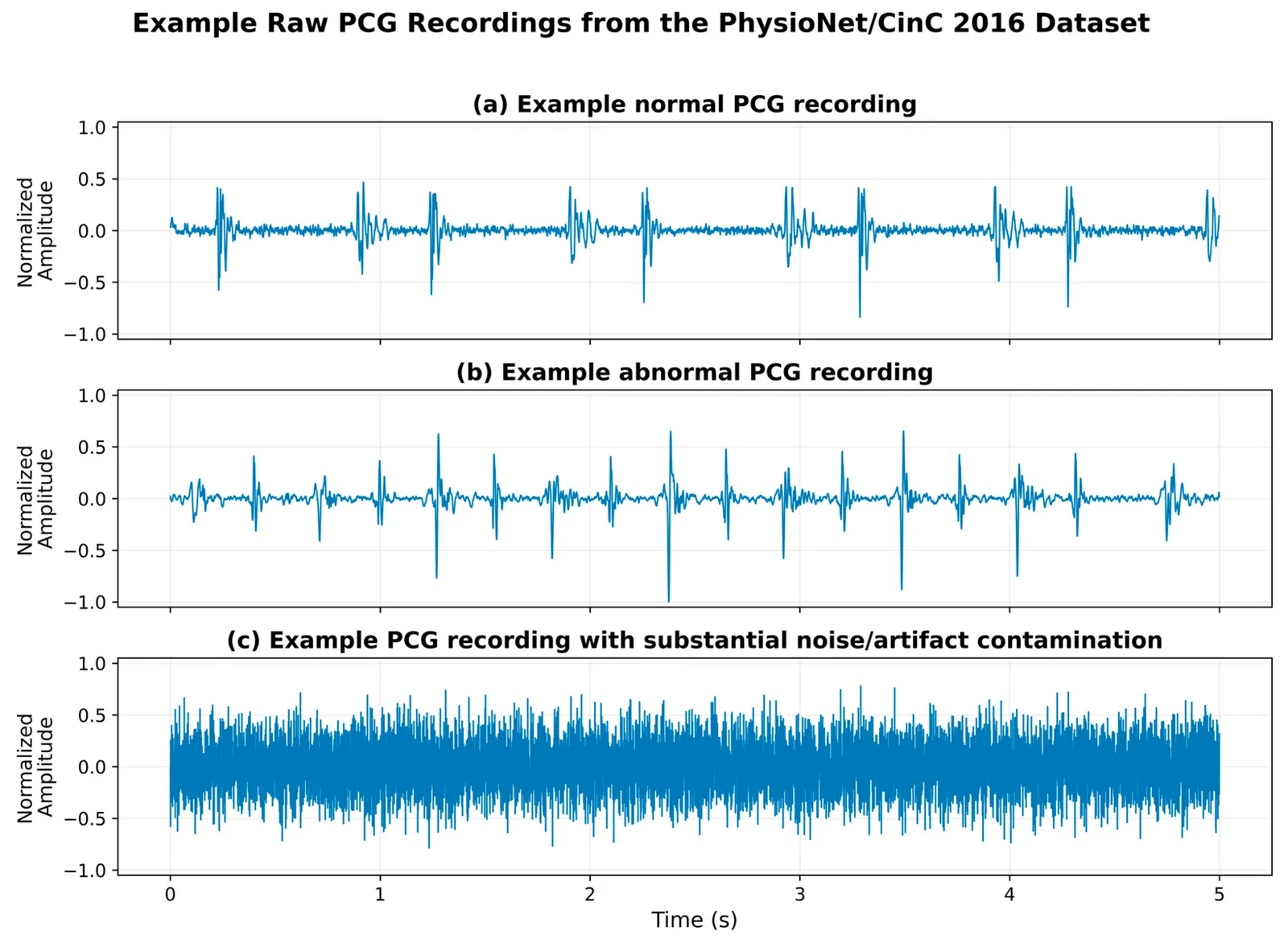
Examples of raw PCG recordings from the PhysioNet/CinC 2016 [2] dataset: (**a**) a normal PCG recording, (**b**) an abnormal PCG recording, and (**c**) a PCG recording exhibiting substantial noise/artifact contamination. Five-second segments are displayed using a common time and normalized-amplitude scale to facilitate visual comparison across several cardiac cycles.

Figure 1 illustrates these characteristics using enlarged five-second segments from the dataset. Figure 1a and Figure 1b show examples of normal and abnormal PCG recordings, respectively, over several cardiac cycles, whereas Figure 1c presents an example recording exhibiting substantial noise/artifact contamination. The examples demonstrate both the morphological variability of raw PCG signals and the potential influence of signal-quality degradation. These characteristics motivate the preprocessing and complementary representation-learning strategy adopted in TriFusion-PCGNet, in which different deterministic transformations of the same underlying PCG signal are jointly analyzed.

Before model evaluation, we performed an explicit duplicate audit between the training and validation directories. Duplicate identity was assessed using both recording identifiers and SHA-256 hashes computed from the original WAV files. All 301 recordings in the validation directory were found to have exact counterparts in the training data: all 301 matched both by recording identifier and by SHA-256 hash. Consequently, the validation directory was excluded entirely from the analytical dataset and was not used for training, model selection, cross-validation, or final evaluation. The resulting analytical dataset therefore consisted exclusively of the 3240 recordings from the six training subsets (2575 normal and 665 abnormal; see Table A1). A complete recording-level manifest and duplicate-audit information are provided in Supplementary Table S1 and the accompanying duplicate-check report.

Reliable explicit subject identifiers were available for 490 recordings corresponding to 106 subjects (see Table A2). Subject identifiers for the remaining recordings were not inferred from filenames or recording identifiers because such inference could not be validated reliably. The complete available subject-to-recording mapping and the subject-disjoint outer-fold assignments are therefore provided in Supplementary Table S2.

## 3. Proposed Methodology

Heart sound analysis is intrinsically difficult due to the non-stationary nature of phonocardiogram (PCG) signals, environmental noise, inter-patient variability, and the complex temporal properties of normal and pathological cardiac events. Many deep learning approaches to PCG analysis rely on a single signal representation, such as the raw waveform or a time–frequency representation. These approaches have demonstrated promising results, but the use of only one signal representation might limit the discriminative power of the model, as different deterministic representations emphasize different signal characteristics of the same PCG recording.

To address this limitation, this work proposes a hybrid deep learning architecture (TriFusion-PCGNet) with multiple branches that exploits three complementary representations of the same PCG recording simultaneously. The proposed framework uses information from the raw PCG signal, the band-pass filtered signal, and the Hilbert envelope signal rather than just extracting features from the raw waveform. Each representation emphasizes different signal characteristics of the same underlying PCG recording, allowing the proposed network to learn complementary feature representations from multiple deterministic views of the same signal.

**Figure 2:**
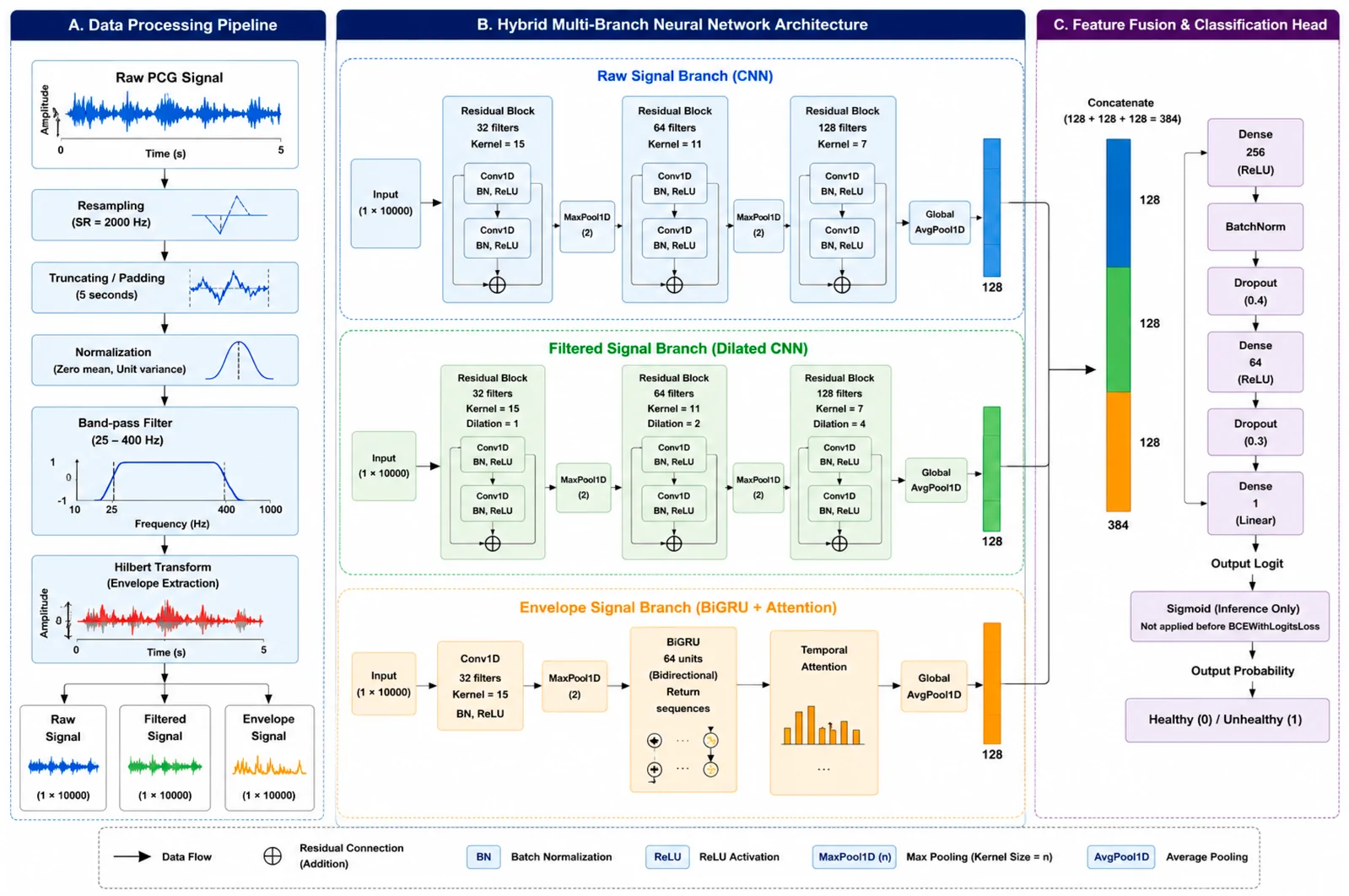
Framework of the proposed approach (TriFusion-PCGNet) architecture.

The framework of the proposed approach is depicted in Figure 2 with three main stages, namely, (A) PCG signal preprocessing, (B) hybrid multi-branch feature extraction, and (C) feature fusion and classification.

In the first stage (see Figure 2A), a series of preprocessing operations is performed on every heart sound recording for signal quality enhancement and multiple signal representations generation suitable for deep feature extraction. First, all recordings are resampled to a common sampling frequency of 2000 Hz and transformed into a fixed-length representation of 5 s by truncation or zero-padding. Amplitude normalization is then applied to suppress recording-dependent intensity variations. A fourth-order Butterworth band-pass filter with cutoff frequencies of 25 and 400 Hz was applied to attenuate low-frequency baseline and motion-related components and high-frequency interference while retaining the frequency range containing the principal heart-sound information. The filter was not intended to eliminate all sources of noise. In particular, power-line interference at 50/60 Hz lies within the selected passband and therefore cannot be specifically suppressed by this band-pass operation. We did not apply a dedicated notch filter because indiscriminate suppression around 50/60 Hz could also remove signal components within the PCG spectrum. Instead, we used the filtered signal as a complementary representation alongside the raw waveform and Hilbert-envelope representation, allowing the network to learn from multiple transformations of the same recording. Finally, the Hilbert transform is applied to obtain the envelope signal, which highlights the temporal distribution of cardiac cycle energy. Thus, each recording provides three synchronized signal representations, namely the raw PCG signal, the band-pass filtered signal and the Hilbert envelope signal.

The second stage (Figure 2B) is the core of the proposed architecture. Three parallel branches for feature extraction are designed to process the three signal representations independently. The first branch utilizes a residual one-dimensional convolutional neural network to process the raw PCG waveform and learn morphological characteristics directly from the original signal. The second branch applies dilated residual convolutions on the filtered signal to increase the receptive field and capture long-range temporal dependencies without substantially increasing the number of learnable parameters. The third branch combines convolutional feature extraction with a bidirectional gated recurrent unit (BiGRU) and a temporal attention mechanism to learn sequential dependencies within the envelope signal and to automatically highlight the most informative cardiac events. The proposed architecture treats these branches as cooperative learners, where each branch can learn complementary feature representations instead of being considered as independent classifiers. The raw signal branch preserves the full waveform morphology, the filtered branch emphasizes noise-suppressed spectral-temporal structures, and the envelope branch captures global energy variations and cardiac cycle dynamics. In the proposed framework, the structural, temporal and energy information are combined into a common representation.

In the final stage (Figure 2C), the feature vectors acquired from the three parallel branches are concatenated to obtain a comprehensive feature representation. Each branch outputs a feature vector of size 128, and the concatenation operation produces a fused feature vector of size 384. The fused representation is then passed through fully connected layers with batch normalization and dropout regularization to learn higher-level discriminative features and avoid overfitting. Finally, a sigmoid activation function estimates the probability of the recording falling into the normal or abnormal class.

The proposed architecture is designed to leverage the complementary characteristics of multiple PCG signal representations, instead of relying on a single input modality. Moreover, the residual learning, dilated convolution, bidirectional recurrent modeling and temporal attention jointly allow the network to learn local morphological patterns, long-term temporal dependencies and energy-based characteristics of heart sounds simultaneously. The integration design aims to improve the discriminative capability of the model under variations in recording characteristics.

The overall workflow of the proposed framework is summarized in the following.

- Signal Acquisition: Raw PCG recordings are acquired from the PhysioNet heart sound database.
- Signal preprocessing: Resampling, conversion to fixed length, normalization, band-pass filtering and Hilbert envelope extraction are sequentially applied.
- Parallel feature extraction: Three specialized deep learning branches process the raw, filtered, and envelope signals independently.
- Feature fusion: The learned feature vectors are concatenated into a unified high-dimensional representation.
- Binary classification: Fully connected layers estimate the probability of a heart sound being normal or abnormal.

In summary, the proposed hybrid architecture fuses multiple complementary signal representations with dedicated branches of deep neural networks, in order to enhance the discriminative power of automatic heart sound classification. Unlike the traditional single-branch CNN-based methods, the proposed framework exploits the waveform morphology, frequency-selective information, temporal dynamics and energy distribution simultaneously, which provides a richer representation of the cardiac acoustic signals for robust binary classification.

### 3.1. Main Contributions of the Proposed Framework

The principal contributions of the proposed framework can be summarized as follows:

A novel multi-representation PCG learning strategy is proposed. Unlike conventional approaches that utilize only the raw phonocardiogram waveform, the proposed framework simultaneously exploits three complementary signal representations, namely the raw PCG signal, the band-pass filtered signal, and the Hilbert envelope signal. This design enables the network to learn morphological, spectral, and energy-related characteristics of heart sounds in a unified architecture. A hybrid multi-branch deep learning architecture is developed. Three specialized feature extraction branches are designed to process different signal representations independently. Each branch is optimized according to the signal characteristics of its corresponding input, allowing complementary feature learning rather than forcing a single network to model all signal properties.

Residual learning, dilated convolution, recurrent temporal modeling, and attention mechanisms are integrated into a unified framework. The proposed architecture combines residual convolutional blocks for stable feature extraction, dilated convolutions for enlarging the receptive field, bidirectional gated recurrent units for sequential modeling, and temporal attention for adaptive emphasis of informative cardiac events. A complementary feature fusion strategy is introduced. Instead of performing independent classification for each signal representation, high-level feature vectors extracted from the three parallel branches are concatenated before the final classification stage. This fusion strategy enables the classifier to exploit complementary information learned from different signal representations of the same PCG recording.

An end-to-end automatic heart sound classification framework is established. The proposed architecture performs signal preprocessing, deep feature extraction, feature fusion, and binary classification within a unified learning framework, eliminating the need for handcrafted feature engineering while maintaining high discriminative capability. Despite its multi-branch design, the proposed framework maintains a relatively compact parameter footprint through one-dimensional operations and global average pooling. The computational analysis therefore characterizes the architecture in terms of its performance–complexity trade-off rather than assuming computational superiority or deployment readiness.

As summarized in Table 1, each branch of the proposed architecture is intentionally designed to process a specific representation of the PCG signal rather than applying the same feature extractor to all inputs. This design philosophy is motivated by the observation that different deterministic signal representations emphasize different characteristics of the same underlying PCG recording. Consequently, specialized feature extractors enable the proposed framework to learn complementary information more effectively than a homogeneous single-branch architecture.

To facilitate reproducibility, the complete layer-level configuration of the proposed TriFusion-PCGNet is summarized in Table 2, including convolutional kernel sizes, dilation rates, strides, padding, pooling operations, residual projections, recurrent and attention dimensions, and classification-head settings.

Unless otherwise specified, all Conv1D layers use a stride of 1. Residual shortcut projections employ 1 × 1 convolutions when the input and output channel dimensions differ. No custom weight initialization was applied, and the default PyTorch 2.1.2 initialization was used for all trainable layers.

### 3.2. PCG Signal Pre-Processing

The quality of phonocardiogram (PCG) recordings plays a critical role in the performance of automatic heart sound classification systems. Raw PCG signals generally exhibit considerable variability due to differences in recording devices, sensor placement, patient-specific cardiac characteristics, respiratory sounds, environmental noise, and motion artifacts. These factors may obscure diagnostically important acoustic events and negatively affect the feature learning capability of deep neural networks. Therefore, an effective preprocessing pipeline is essential to improve signal consistency while preserving clinically relevant cardiac information.

As illustrated in Figure 2A, each heart sound recording undergoes a sequence of preprocessing operations before being presented to the proposed hybrid deep learning architecture. These operations consist of resampling, fixed-length representation, amplitude normalization, Butterworth band-pass filtering, and Hilbert envelope extraction. The objective of this preprocessing stage is not only to suppress unwanted signal components but also to generate multiple complementary representations of the same cardiac cycle that can later be processed by specialized feature extraction branches.

Unlike conventional approaches that utilize only the original PCG waveform, the proposed framework simultaneously employs three synchronized signal representations, namely the raw PCG signal, the band-pass filtered signal, and the Hilbert envelope signal. This multi-representation strategy enables the proposed architecture to capture morphological, temporal, and energy-related information from complementary perspectives, thereby improving feature diversity and classification robustness.

The PCG recordings contained in the PhysioNet database may originate from different acquisition systems and therefore may have different sampling frequencies. To ensure a uniform input representation, every recording is resampled to a common sampling frequency of 2000 Hz. This selection offers sufficient temporal resolution to preserve clinically important heart sound components while simultaneously reducing computational complexity compared to higher sampling rates. Since the dominant frequency content of normal and abnormal heart sounds is typically below 400 Hz, the selected sampling frequency fully satisfies the Nyquist criterion. Deep neural networks require inputs of identical dimensions. However, the duration of PCG recordings varies among patients. Therefore, all recordings are converted into a fixed-length representation of 10,000 samples (T = 5 s). If the recording is shorter than five seconds, zero-padding is applied. Otherwise, the first five-second segment is retained. This operation ensures identical input dimensions for every sample while preserving the temporal integrity of the recorded cardiac cycle.

Heart sound recordings may exhibit substantial amplitude variations due to differences in stethoscope sensitivity, sensor position, and recording conditions. To eliminate these variations, each signal is independently normalized according to Equation (1).

(1) $$\tilde{x}\left(n\right)=\frac{x_{f}(n)}{max\left|x_{f}\left(n\right)\right|+\varepsilon }$$

where *ε* (10^−8^) is a small constant to avoid numerical instabilities. This normalization maps each recording into the interval [−1, 1] so that the neural network can concentrate on waveform morphology rather than on absolute signal amplitude.

Amplitude normalization reduces inter-recording variability, but PCG signals still contain low-frequency motion artifacts and high-frequency environmental noise. Hence, a fourth-order Butterworth band-pass filter is used with cut-off frequencies of 25 ≤ f ≤ 400 Hz. A fourth-order Butterworth filter was preferred because its maximally flat passband preserves the morphology of heart sounds while effectively attenuating undesired frequency components. Compared with filters exhibiting passband ripple, the Butterworth response minimizes waveform distortion, which is particularly important for subsequent deep feature learning. The passband selected retains S1 heart sound, S2 heart sound, systolic murmurs, and diastolic murmurs, while attenuating low-frequency motion-related components and high-frequency interference. The signal ($\hat{x})$ is again normalized as in Equation (1) after filtering. The second normalization is used to compensate for the amplitude attenuation introduced by the filtering operation.

Although the filtered signal preserves most clinically relevant frequency components, temporal energy variations are not explicitly represented. Therefore, the analytic signal is computed using the Hilbert transform [32]. The Hilbert transform is defined as in Equation (2).

(2) $$H\left\{\hat{x}\left(t\right)\right\}=\frac{1}{\pi }PV\int_{-\infty }^{+\infty }\frac{\hat{x}\left(\tau \right)}{t-\tau }d\tau$$

where *PV* denotes the Cauchy principal value. The corresponding analytic signal is provided in Equation (3). The envelope signal is derived from Equation (3), as shown in Equation (4). Finally, the envelope signal is normalized according to Equation (5).

(3) $$z\left(t\right)=\hat{x}\left(t\right)+jH\{\hat{x}\left(t\right)\}$$

(4) $$E\left(t\right)=\left|z(t)\right|=\sqrt{{\hat{x}}^{2}\left(t\right)+H^{2}\{\hat{x}\left(t\right)\}}$$

(5) $$\tilde{E}\left(t\right)=\frac{E(t)}{max\left|E(t)\right|+\varepsilon }$$

The Hilbert envelope emphasizes the temporal energy distribution of heart sounds by highlighting the locations of S1, S2, systolic intervals, diastolic intervals, and pathological murmurs. Consequently, the envelope provides complementary information that cannot be easily extracted from the waveform alone [33].

Following the preprocessing stage, three synchronized signal representations are obtained for every PCG recording:

(6) $$X=\{\tilde{x},\;\hat{x},\tilde{E}\}$$

where $\tilde{x}$ represents the normalized raw PCG signal, $\hat{x}$ denotes the normalized band-pass filtered signal, and $\tilde{E}$ corresponds to the normalized Hilbert envelope. These three complementary representations constitute the inputs of the proposed hybrid multi-branch deep learning framework shown in Figure 2B. Each representation emphasizes different signal characteristics of the same underlying PCG recording, enabling the subsequent feature-extraction branches to learn complementary representations from multiple deterministic views of the same signal.

### 3.3. Hybrid Multi-Branch Feature Extraction Network

Following the preprocessing stage, each PCG recording is represented by three synchronized signal modalities, namely the normalized raw PCG signal, the normalized band-pass filtered signal, and the normalized Hilbert envelope. Although these signals originate from the same cardiac recording, each representation emphasizes different signal characteristics of the same underlying PCG recording. Consequently, employing a single feature extraction network for all representations may not fully exploit their complementary information. To address this limitation, a hybrid multi-branch deep neural network is proposed, as illustrated in Figure 2B. Instead of forcing a single backbone to process heterogeneous signal representations, three specialized feature extraction branches are constructed. Each branch is independently optimized according to the characteristics of its corresponding input representation.

Unlike homogeneous multi-input CNN architectures, the proposed framework adopts different deep learning modules for different signal modalities. This design philosophy is motivated by the observation that the diagnostic information contained in waveform morphology, denoised acoustic structures, and temporal energy distribution exhibits significantly different statistical properties. Therefore, assigning specialized feature extractors enables each branch to learn more discriminative representations before feature fusion. Overall, the proposed network consists of three parallel feature extraction branches followed by a shared feature fusion and classification module [34].

#### 3.3.1. Raw Signal Branch

The first branch is meant to process the normalized raw PCG waveform. Since the raw signal retains the full acoustic morphology of heart sounds, the aim of this branch is to learn local waveform features directly from the original recording. This branch consists of 3 residual 1D convolutional blocks with 32, 64 and 128 filters, respectively. We choose kernel sizes of 15, 11 and 7 to allow the network to transition from coarse-scale acoustic patterns to fine-scale local structures. Each residual block consists of two consecutive 1D convolution layers with batch normalization and ReLU activation.

Instead of learning the complete mapping directly, residual learning estimates only the residual function *F*(*X*), which is combined with the identity mapping as *Y* = *F*(*X*) + *X*. Residual connections significantly improve gradient propagation during backpropagation and reduce performance degradation in deeper networks [35].

The raw PCG waveform contains complete morphological information regarding S1, S2, clicks, snaps, and murmurs. Therefore, residual CNNs are employed to extract hierarchical morphological representations while preserving low-level acoustic structures through identity shortcuts. The gradually decreasing kernel sizes enable the network to learn both coarse cardiac events and fine local waveform details.

#### 3.3.2. Band-Pass Filtered Signal Branch

Although the raw waveform preserves all acoustic information, residual background noise may still affect feature extraction. Therefore, a second branch is constructed using the band-pass filtered PCG signal. The overall architecture is similar to the first branch but replaces conventional convolutions with dilated residual convolutions [36].

For a dilation factor *r*, the dilated convolution is defined as shown in Equation (7).

(7) $$Y\left(i\right)=\sum_{k=1}^{K}W\left(k\right)X(i-rk)$$

where r = 1, 2, 4 is successive residual blocks. Increasing the dilation factor enlarges the receptive field without increasing the number of learnable parameters. Consequently, the network simultaneously captures local waveform characteristics and longer temporal dependencies associated with pathological murmurs. Global Average Pooling is used again to create a 128-dimensional feature vector.

Band-pass filtering reduces irrelevant frequency components before deep feature extraction. Combining this denoised representation with dilated convolutions allows the network to capture longer cardiac contexts while remaining computationally efficient.

#### 3.3.3. Hilbert Envelope Branch

Unlike the previous branches, the envelope representation primarily describes temporal energy variations rather than detailed waveform morphology. Therefore, this branch adopts a hybrid sequential architecture consisting of Conv1D, BiGRU, and temporal attention. Initially, Conv1D extracts local energy transitions; subsequently, BiGRU models temporal dependencies in both forward and backward directions [37]. The update gate of the GRU is computed as

(8) $$z_{t}=\sigma \left(W_{z}x_{t}+U_{z}h_{t-1}\right),$$

where $x_{t}$ denotes the input vector at time step t, $h_{t-1}$ represents the hidden state from the previous time step, $W_{z}$ and are learnable weight matrices, and σ(.) denotes the sigmoid activation function. The update gate determines how much information from the previous hidden state should be retained in the current memory. Similarly, the reset gate is calculated as

(9) $$r_{t}=\sigma \left(W_{r}x_{t}+U_{r}h_{t-1}\right),$$

where $W_{r}$ and $U_{r}$ denote the trainable parameters associated with the reset gate. The reset gate controls the amount of historical information that should be forgotten before computing the candidate hidden representation.

Using the update and reset gates, the hidden state is updated according to

(10) $$h_{t}=\left(1-z_{t}\right)\tilde{h_{t}}+z_{t}h_{t-1},$$

where $\tilde{h_{t}}$ denotes the candidate hidden state generated by the GRU cell, $h_{t-1}$ represents the hidden state from the previous time step, and $h_{t}$ denotes the updated hidden representation at time step *t*. The update gate $z_{t}$ controls the contribution of the previous hidden state and the candidate hidden state to the updated representation. This gated memory mechanism enables the network to preserve long-range temporal dependencies while alleviating the vanishing-gradient problem commonly encountered in conventional recurrent neural networks.

Although the BiGRU produces contextual representations for every time step, not all cardiac events contribute equally to the final classification. Therefore, a temporal attention mechanism is employed to automatically identify diagnostically informative regions [38].

The attention weights are computed using the Softmax function:

(11) $$\alpha_{t}=\frac{{exp}^{e_{t}}}{\sum_{j}exp\left(e_{j}\right)}\;,$$

The envelope characterizes temporal variations in signal amplitude, while the BiGRU models sequential dependencies and the temporal attention mechanism assigns different weights to temporal features according to their contribution to the learned representation.

Where $\alpha_{t}$ denotes the normalized attention coefficient assigned to the t temporal feature and $e_{t}$ represents the corresponding attention score. The unnormalized attention score is calculated as

(12) $$e_{t}=v^{T}tanh\left(Wh_{t}+b\right),$$

where $h_{t}$ is the hidden feature generated by the BiGRU, W denotes the learnable projection matrix, b is the bias vector, v is the trainable attention vector, and *tanh*(*⋅*) is the hyperbolic tangent activation function. This operation transforms the sequential hidden representations into scalar attention scores that reflect the diagnostic relevance of each temporal location [24].

Finally, the context vector is obtained as the weighted sum of all hidden representations,

(13) $$c=\sum_{t}\alpha_{t}h_{t},$$

where c is the final envelope feature representation, which is fed into the subsequent feature fusion stage. In this formulation, hidden states associated with diagnostically relevant cardiac events obtain higher attention weights and thus contribute more heavily to the final feature vector.

The proposed envelope branch utilizes bidirectional temporal modeling and adaptive attention weighting jointly, as shown by Equations (8)–(13). The BiGRU learns long-term sequential dependencies over the entire cardiac cycle, and the Temporal Attention mechanism learns to automatically focus on informative regions corresponding to S1, S2, systolic murmurs, and diastolic murmurs. The envelope branch produces a discriminative high-level representation that complements the morphological information extracted by the convolutional branches.

## 4. Experimental and Ablation Studies

The experiments were implemented in PyTorch 2.1.2 with CUDA 12.1 (Meta Platforms, Inc., Menlo Park, CA, USA) and executed using an NVIDIA GeForce RTX 4090 GPU (NVIDIA Corporation, Santa Clara, CA, USA). Computational complexity was quantified using the number of trainable parameters and FLOPs, while peak GPU memory consumption and inference time for a 5 s PCG input were measured to characterize the practical computational requirements of the models.

The Adam optimizer was chosen due to its adaptive learning rate and its proven ability to train deep neural networks. Adam was selected because its adaptive parameter-wise learning rates can facilitate stable optimization of architectures that combine convolutional, recurrent, and attention-based components. The initial learning rate was set to 1 × 10^−4^, and the mini-batch size was 16. All models were trained for a maximum of 150 epochs. We used Binary Cross-Entropy with Logits Loss (BCEWithLogitsLoss) as the objective function since it combines the sigmoid operation and binary cross-entropy in a numerically stable formulation, which is suitable for binary classification.

Although the dataset is imbalanced toward normal recordings, the standard BCEWithLogitsLoss was retained in the present study to provide a simple and consistent optimization objective without introducing additional class-dependent weighting or loss-specific hyperparameters. This choice also allowed the contribution of the proposed multi-representation architecture to be evaluated without confounding it with specialized imbalance-handling losses. Nevertheless, the class imbalance may affect minority-class recognition, and the lower sensitivity relative to specificity observed in the experiments indicates that abnormal recordings remain more difficult to identify. Therefore, the use of standard BCEWithLogitsLoss should not be interpreted as evidence that class imbalance has no effect on model performance.

We used a strictly separated model-selection and evaluation procedure for the stratified 10-fold cross-validation experiment. In each outer cross-validation iteration, one fold was held out as the outer test fold only, and the other nine folds were the outer training set. The outer training set was split into an internal training set and an internal validation set. The model optimization was performed only on the internal training subset, while the internal validation subset was used for epoch selection and model checkpointing. The model was tested on the internal validation subset after each epoch, and model parameters with the best internal validation accuracy were saved. Crucially, the outer test fold was not used in training, epoch selection, checkpoint selection, or any other model selection process.

After the best model checkpoint had been chosen solely based on the internal validation subset, the best model was tested once on the corresponding untouched outer test fold. Final performance indicators reported for each fold (accuracy, precision, sensitivity, specificity, F1-score, AUC, and confusion matrix) were all calculated strictly from predictions on this outer test fold. This procedure was repeated for all ten outer folds, so that each sample was used once in the independent final evaluation. The model parameters were reinitialized before each outer fold to ensure independent training processes. Finally, predictions from all ten untouched outer test folds were combined to yield the overall cross-validation confusion matrix and performance estimates. This strict separation of internal validation from outer-fold testing prevents the final evaluation data from influencing model selection and thus reduces optimistic bias.

**Figure 3:**
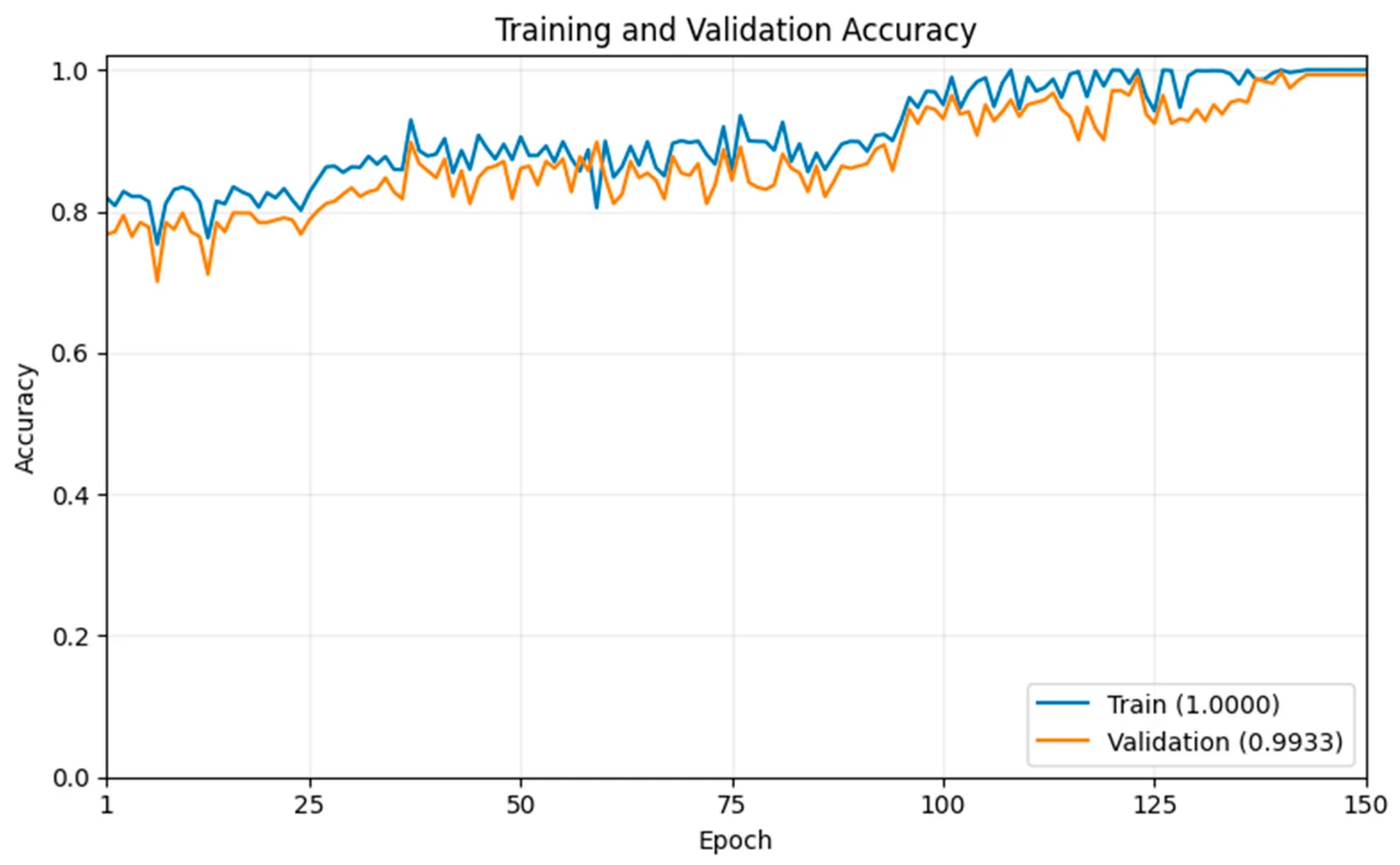
Accuracy change graph of the TriFusion-PCGNet model (Separation of training and validation data in the database).

Figure 3 shows the training and validation accuracy of the proposed TriFusion-PCGNet with the original train-validation partition of the PhysioNet/CinC 2016 dataset. Both curves increase rapidly during the early training epochs and subsequently approach a plateau. Validation accuracy shows moderate fluctuations during intermediate epochs but remains high toward the end of training. The training and validation accuracies after 150 epochs are 100.0% and 99.33%, respectively, indicating that the proposed architecture is able to learn highly discriminative representations from PCG signals. The small gap between the two curves indicates similar optimization behavior on the training and validation subsets under this fixed split; however, these results should not be interpreted as evidence of patient-independent generalization.

**Figure 4:**
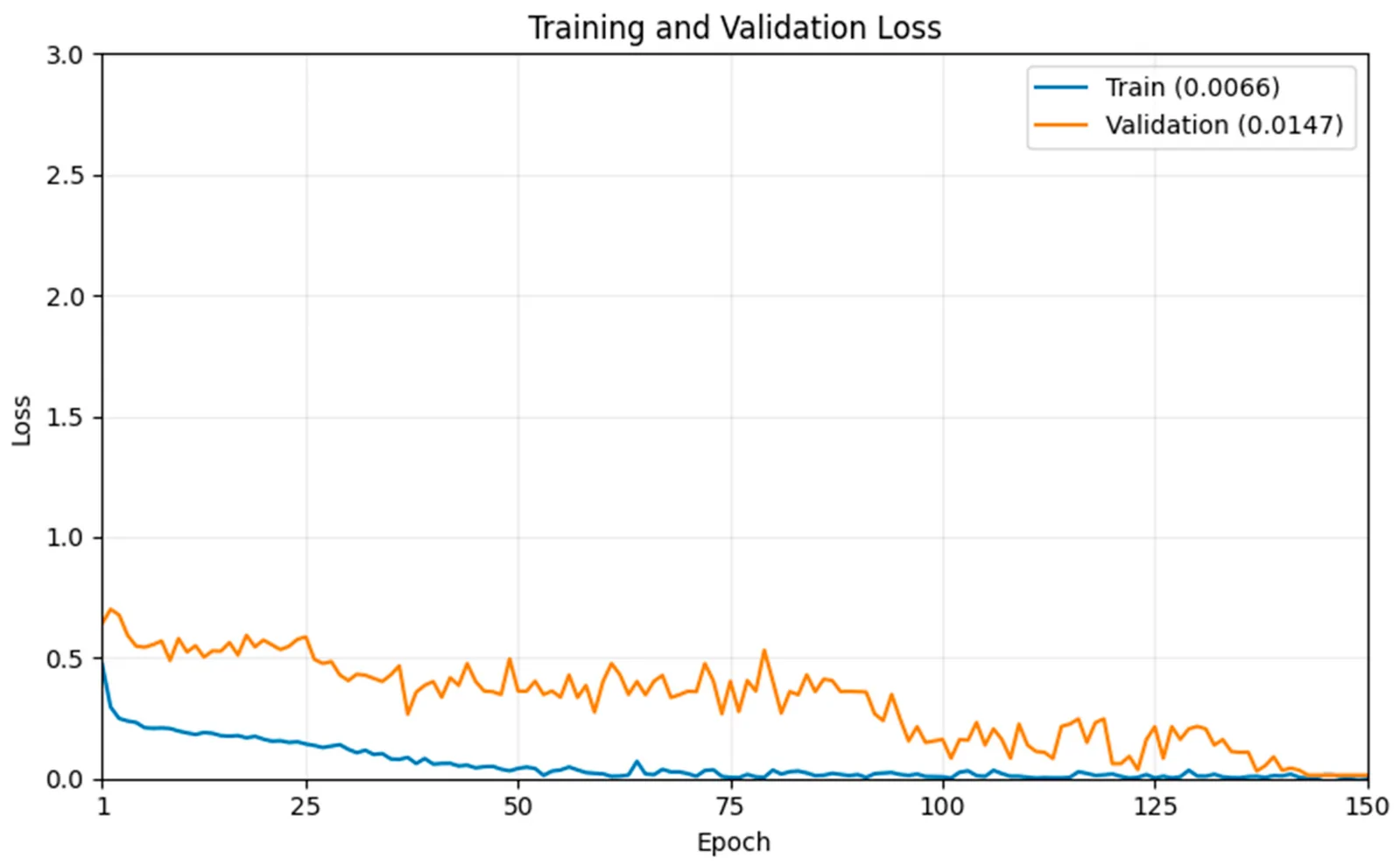
Loss change graph of the TriFusion-PCGNet model (Separation of training and validation data in the database).

Figure 4 shows the corresponding curves of training and validation loss using the same train-validation partition. Training and validation losses generally decrease over the course of optimization, although the validation curve exhibits temporary fluctuations. At the end of training, the training and validation losses reached 0.0066 and 0.0147, respectively. The low training and validation losses indicate successful optimization under this fixed train–validation split; however, they do not establish generalization to unseen subjects.

**Figure 5:**
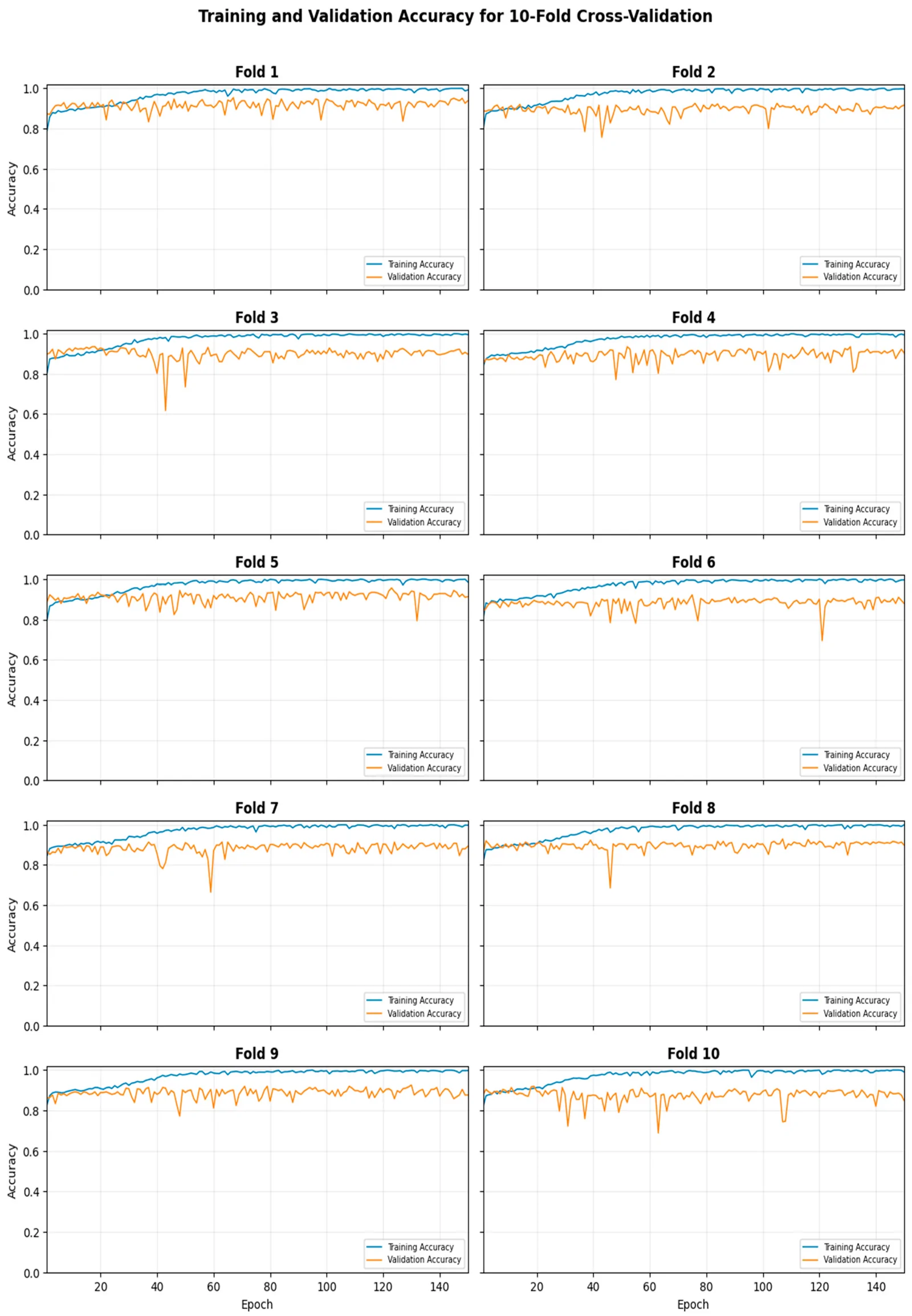
Training and internal-validation accuracy curves of TriFusion-PCGNet across the 10 outer cross-validation folds.

Figure 5 shows the training and internal-validation accuracy curves for all ten outer folds of the stratified 10-fold cross-validation experiment. In each outer-fold iteration, the training curves were obtained from the internal training subset, while the validation curves correspond to the internal validation subset used exclusively for model selection and checkpointing. The outer test fold was kept completely isolated during this process and was not used to select the best epoch or model checkpoint. The learning curves generally show rapid improvement during the early epochs followed by stable optimization. Variations in internal-validation accuracy across folds reflect differences among the internal validation subsets. Final classification performance was determined separately by evaluating the selected model once on the corresponding untouched outer test fold.

**Figure 6:**
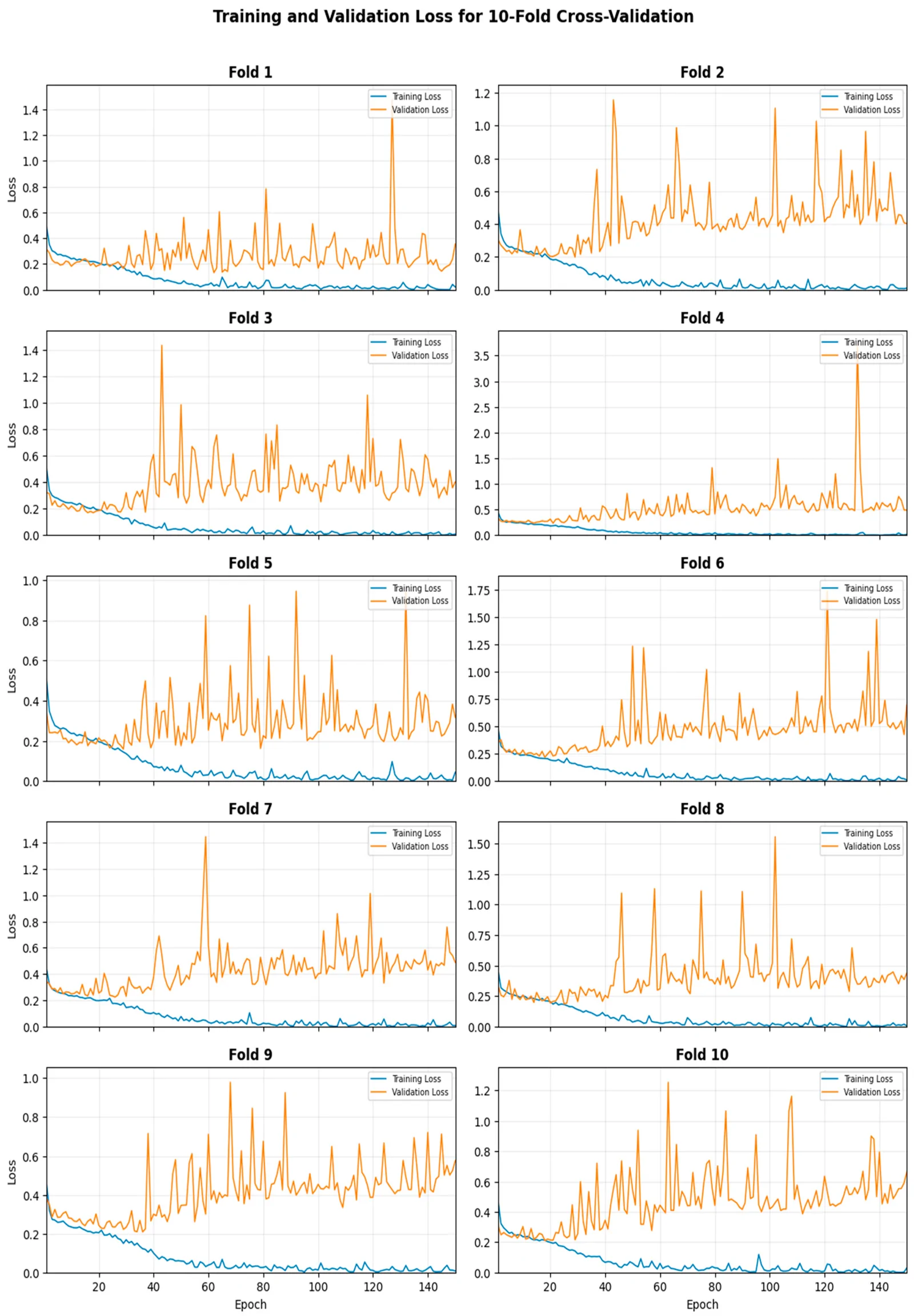
Training and internal-validation loss curves of TriFusion-PCGNet across the 10 outer cross-validation folds.

Figure 6 shows the corresponding training and internal-validation loss curves for all ten outer-fold iterations. The training loss generally decreases toward low values, whereas the internal-validation loss exhibits some fold-dependent fluctuations during optimization. These internal-validation curves were used only to monitor the optimization process and select the model checkpoint. Importantly, the outer test fold was not involved in epoch selection or checkpoint selection. After the optimal checkpoint had been selected using the internal validation subset, the model was evaluated once on the corresponding untouched outer test fold to obtain the final performance metrics.

**Figure 7:**
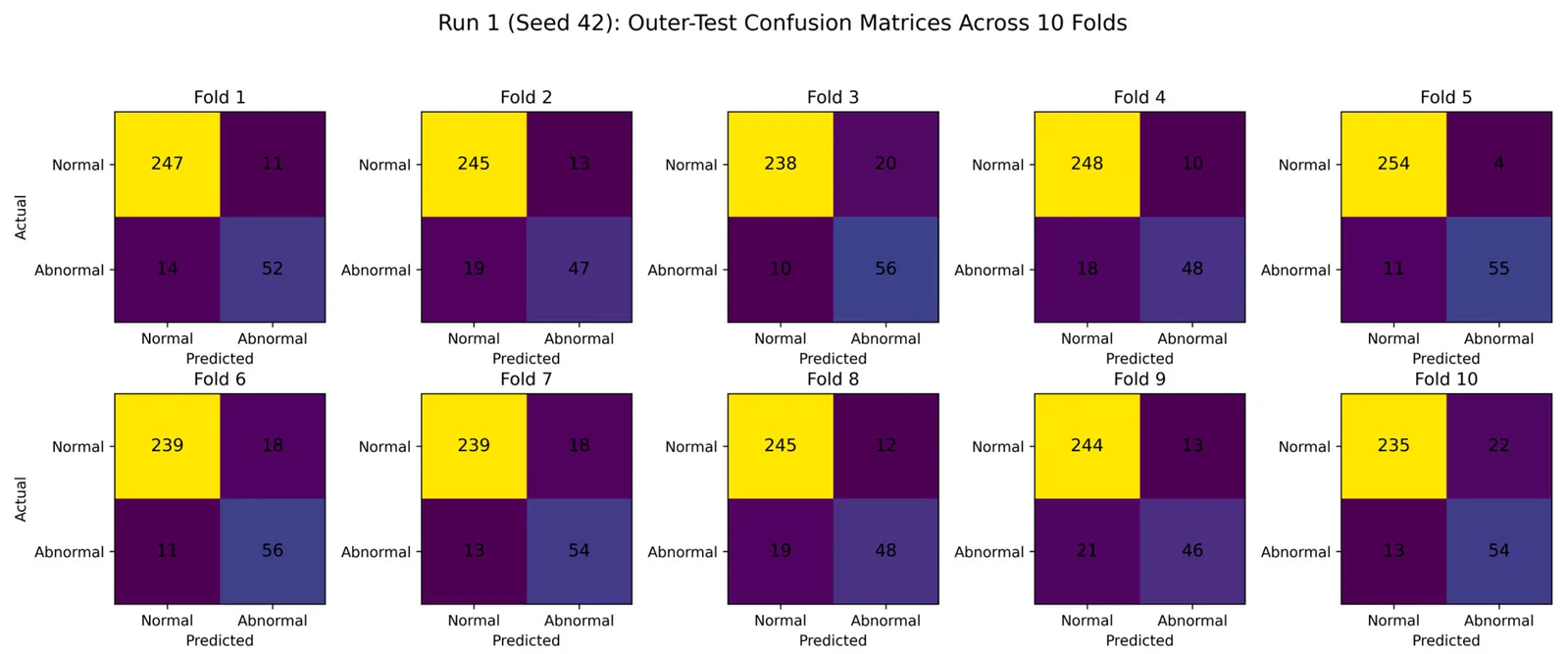
Outer-test confusion matrices for the ten folds of Run 1 (seed 42) under the strictly separated record-level 10-fold cross-validation protocol. Each matrix represents predictions obtained exclusively from the corresponding untouched outer test fold.

**Figure 8:**
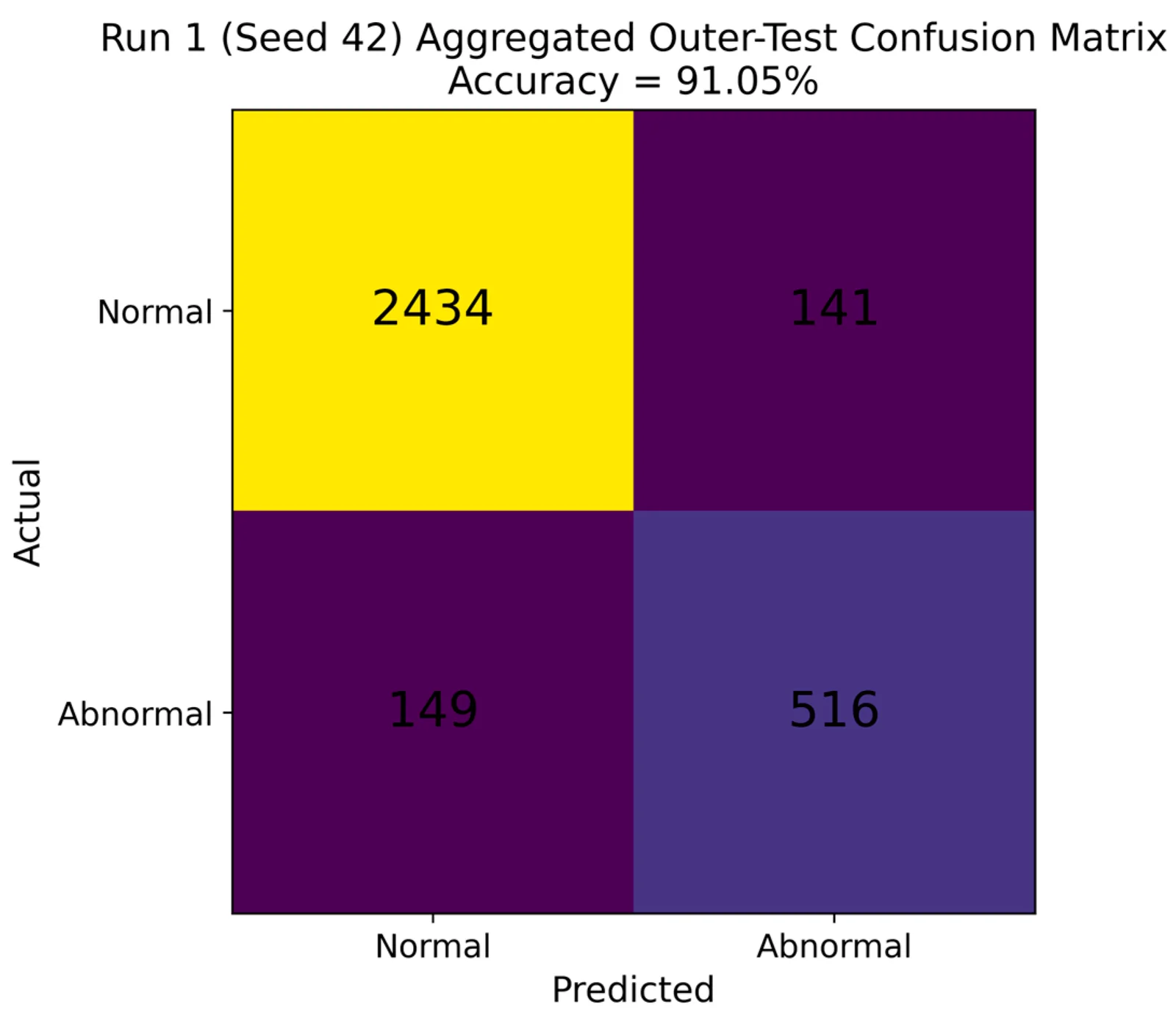
Aggregated outer-test confusion matrix for Run 1 (seed 42), obtained by combining the predictions from all ten outer test folds shown in Figure 7. The aggregated matrix contains 2434 true-normal, 141 false-abnormal, 149 false-normal, and 516 true-abnormal predictions, corresponding to an accuracy of 91.05%, consistent with the Run 1 result reported in Table 5.

Figure 7 presents the fold-specific confusion matrices obtained from the ten untouched outer test folds of Run 1 (seed 42) under the stratified record-level 10-fold cross-validation protocol. Each confusion matrix represents the final evaluation on the corresponding outer test fold after model selection and checkpointing had been completed using only the associated internal training and validation subsets. The outer test recordings were therefore not involved in model training, epoch selection, or checkpoint selection. Across the ten folds, TriFusion-PCGNet correctly classified the majority of both normal and abnormal recordings, although the numbers of false-positive and false-negative predictions varied across folds. These fold-specific matrices correspond to the same Run 1 (seed 42) experiment whose aggregated confusion matrix is presented in Figure 8.

Table 3 presents the fold-wise performance of TriFusion-PCGNet using the strictly separated stratified record-level 10-fold cross-validation procedure with three independent random initializations. The term “strictly separated” refers to the separation of the outer test recordings from training, internal validation, and model selection; it does not imply subject-disjoint partitioning. The same outer-fold partitions were shared between the independent runs. The initialization of the models and stochastic training were repeated with random seeds. The values are reported as mean ± standard deviation over the three runs for each outer test fold. Besides the conventional classification measures, PR-AUC, balanced accuracy and Matthews correlation coefficient (MCC) were added to achieve more comprehensive evaluation under class imbalance. The mean test accuracy across the outer folds was between 89.30% and 93.52%, and ROC-AUC was between 94.37% and 97.42%. Fold 5 had the highest mean test accuracy (93.52%), balanced accuracy (88.60%), MCC (0.796) and ROC-AUC (97.42%) and Fold 10 had the highest mean sensitivity (82.09%). The specificity was high in all the folds, ranging from 91.18% to 96.90%. While sensitivity was more variable than specificity when considering a fold dependence, the results demonstrate that TriFusion-PCGNet retains discriminative ability across held-out record-level outer test partitions and stochastic model initializations.

Table 4 presents the overall performance obtained by aggregating the untouched outer-test predictions within each independent run and subsequently summarizing the three run-level results as mean ± standard deviation and 95% confidence intervals. TriFusion-PCGNet achieved an overall test accuracy of 90.87 ± 0.17%, with a 95% confidence interval of 90.45–91.30%. The corresponding ROC-AUC and PR-AUC were 95.34 ± 0.30% and 82.43 ± 0.65%, respectively. Considering the class imbalance, the model achieved a balanced accuracy of 85.71 ± 0.70% and an MCC of 0.719 ± 0.007. The relatively narrow confidence intervals for overall accuracy and ROC-AUC indicate limited variability in these metrics across the three independent runs. The difference between sensitivity (76.94 ± 1.68%) and specificity (94.47 ± 0.31%) nevertheless indicates that the model identifies normal recordings more consistently than abnormal recordings. Therefore, the additional class-imbalance-aware metrics provide a more informative characterization of model performance than overall accuracy alone.

Figure 8 presents the aggregated confusion matrix obtained by combining the predictions from all ten untouched outer test folds of Run 1 (seed 42). TriFusion-PCGNet correctly classified 2434 normal recordings and 516 abnormal recordings, while 141 normal recordings were misclassified as abnormal and 149 abnormal recordings were misclassified as normal. Thus, 2950 of the 3240 recordings were correctly classified, corresponding to an accuracy of 91.05%, which is consistent with the Run 1 result reported in Table 5. Figure 7 and Figure 8 therefore present the fold-specific and aggregated results, respectively, from the same experimental run (Run 1, seed 42). The primary overall performance estimates remain the three-run mean ± standard deviation values reported in Table 4, including an accuracy of 90.87 ± 0.17% and a ROC-AUC of 95.34 ± 0.30%.

**Table 3:** Performance metrics of TriFusion-PCGNet on the untouched outer test folds of the stratified 10-fold cross-validation for three independent runs.

Fold	Train Acc. (%)	Test Acc. (%)	Precision (%)	Sensitivity (%)	Specificity (%)	F1 (%)	ROC-AUC (%)	PR-AUC (%)	Bal. Acc. (%)	MCC
1	99.21 ± 0.31	92.59 ± 0.82	83.26 ± 5.88	80.30 ± 4.01	95.74 ± 1.94	81.57 ± 1.21	97.12 ± 0.22	88.53 ± 2.88	88.02 ± 1.13	0.771 ± 0.019
2	97.85 ± 1.68	91.15 ± 1.08	82.32 ± 4.55	72.22 ± 0.87	95.99 ± 1.18	76.91 ± 2.37	95.28 ± 0.97	82.95 ± 4.07	84.11 ± 0.97	0.717 ± 0.033
3	99.50 ± 0.36	90.53 ± 0.64	76.92 ± 4.82	77.27 ± 6.94	93.93 ± 1.99	76.83 ± 1.88	95.93 ± 1.03	86.00 ± 1.02	85.60 ± 2.59	0.711 ± 0.023
4	99.63 ± 0.13	91.15 ± 0.36	80.23 ± 2.77	75.25 ± 2.31	95.22 ± 0.98	77.61 ± 0.46	94.37 ± 0.71	78.22 ± 3.26	85.24 ± 0.71	0.722 ± 0.007
5	99.02 ± 0.53	**93.52 ± 1.72**	87.01 ± 5.41	80.30 ± 5.25	96.90 ± 1.40	83.46 ± 4.49	97.42 ± 0.90	86.98 ± 2.87	88.60 ± 2.87	0.796 ± 0.055
6	96.95 ± 3.93	89.61 ± 1.55	75.75 ± 0.78	73.13 ± 10.45	93.90 ± 0.81	74.18 ± 5.60	94.87 ± 0.83	82.26 ± 0.80	83.52 ± 4.84	0.679 ± 0.063
7	98.12 ± 2.00	89.81 ± 1.07	74.10 ± 2.92	78.11 ± 2.28	92.87 ± 0.98	76.04 ± 2.32	95.02 ± 0.32	82.54 ± 1.03	85.49 ± 1.42	0.696 ± 0.030
8	99.30 ± 0.54	91.05 ± 0.82	77.85 ± 2.81	79.60 ± 6.89	94.03 ± 1.37	78.54 ± 2.80	96.18 ± 0.53	83.23 ± 4.96	86.82 ± 2.91	0.730 ± 0.032
9	96.06 ± 4.01	90.02 ± 2.06	78.54 ± 5.07	71.14 ± 5.65	94.94 ± 1.17	74.65 ± 5.34	94.89 ± 0.24	79.56 ± 0.50	83.04 ± 3.37	0.686 ± 0.066
10	99.28 ± 0.53	89.30 ± 0.78	70.96 ± 3.06	82.09 ± 2.59	91.18 ± 1.57	76.05 ± 0.94	95.66 ± 0.07	84.32 ± 1.25	86.63 ± 0.63	0.696 ± 0.012

**Table 4:** Mean ± SD of the three performance values obtained after combining all outer-test predictions for three independent runs.

Metric	Mean ± SD	95% CI
Test Accuracy	90.87 ± 0.17%	90.45–91.30%
Precision	78.25 ± 0.66%	76.62–79.88%
Sensitivity	76.94 ± 1.68%	72.78–81.11%
Specificity	94.47 ± 0.31%	93.69–95.25%
F1-score	77.58 ± 0.66%	75.94–79.22%
ROC-AUC	95.34 ± 0.30%	94.59–96.09%
PR-AUC	82.43 ± 0.65%	80.81–84.05%
Balanced Accuracy	85.71 ± 0.70%	83.96–87.46%
MCC	0.719 ± 0.007	0.701–0.737

Table 5 further reports the run-level results obtained from the three independent random initializations (seeds 42, 123, and 2024). Test accuracy varied only from 90.71% to 91.05%, while ROC-AUC ranged from 95.04% to 95.65%. Similarly, PR-AUC values ranged from 81.70% to 82.97%. Balanced accuracy was 86.06%, 86.17%, and 84.90% for seeds 42, 123, and 2024, respectively, whereas the corresponding MCC values were 0.724, 0.721, and 0.711. These results directly quantify the stochastic variability of the proposed framework and show that the overall performance remains relatively consistent across different random initializations.

**Table 5:** Mean ± SD of the three performance values obtained after combining all outer-test predictions for each independent run.

Seed	Accuracy	ROC-AUC	PR-AUC	Balanced Acc.	MCC
42	91.05%	95.04%	82.62%	86.06%	0.724
123	90.86%	95.65%	82.97%	86.17%	0.721
2024	90.71%	95.34%	81.70%	84.90%	0.711
Mean ± SD	90.87 ± 0.17%	95.34 ± 0.30%	82.43 ± 0.65%	85.71 ± 0.70%	0.719 ± 0.007

**Figure 9:**
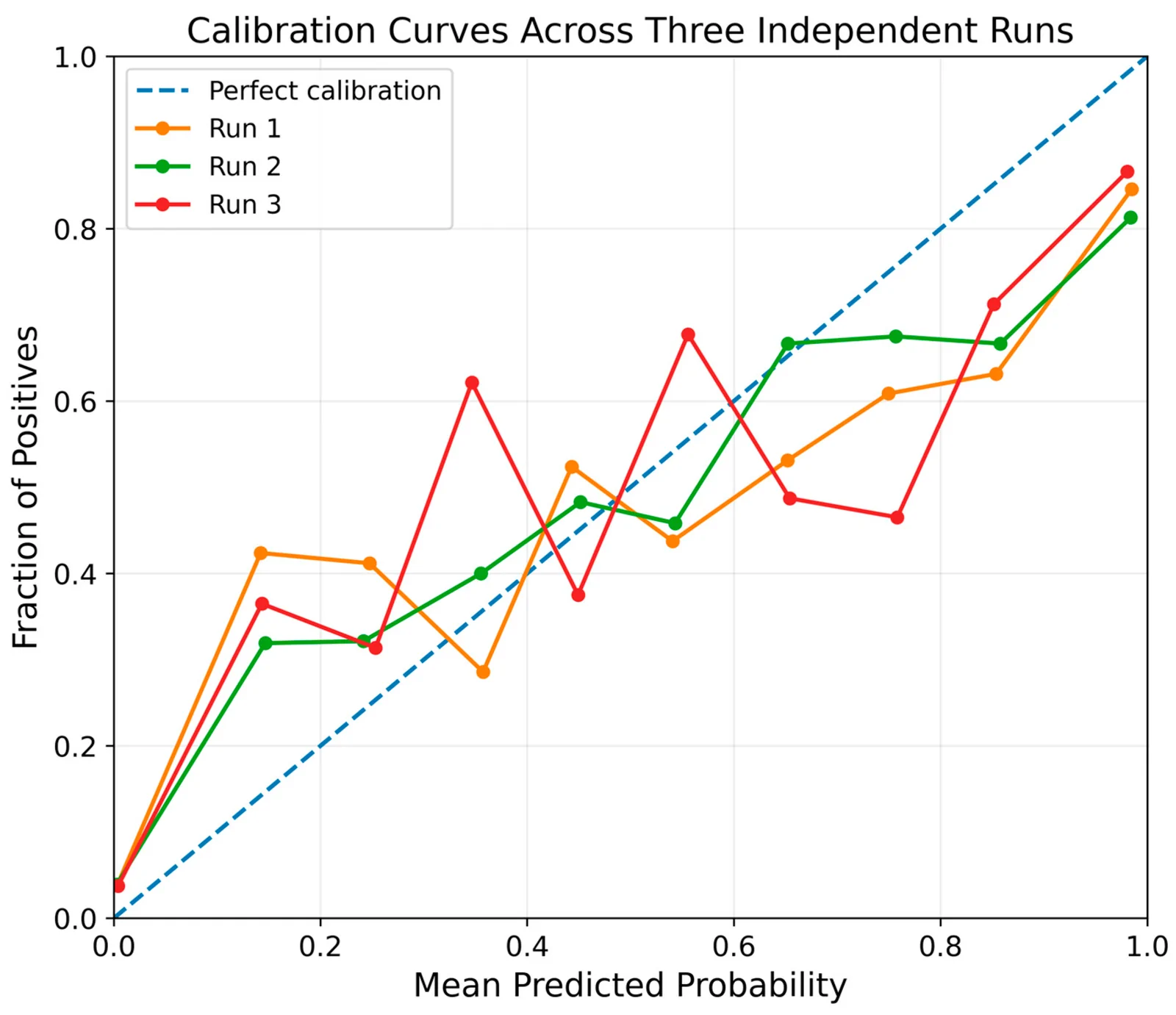
Calibration curves of TriFusion-PCGNet across three independent random initializations. The diagonal dashed line represents perfect calibration, whereas each curve represents the relationship between predicted probabilities and observed positive-class frequencies for an independent run.

Calibration analysis was performed to assess the reliability of the predicted probabilities. The three independent runs yielded ECE values of 0.0637, 0.0640, and 0.0607, corresponding to a mean ECE of 0.0628 ± 0.0018 (Table 6). The corresponding Brier scores were 0.0748, 0.0776, and 0.0752, with a mean of 0.0759 ± 0.0015. The limited variation across runs indicates consistent probabilistic behavior across random initializations. Figure 9 shows the corresponding calibration curves. Although deviations from the ideal diagonal are present in some probability ranges, the three runs exhibit broadly similar calibration patterns. These findings complement the discrimination metrics by providing a quantitative assessment of probability calibration.

Overall, the experimental results demonstrate that TriFusion-PCGNet has stable discriminative performance on the independent outer test folds and under different stochastic initializations. The three-run analysis, together with the reported confidence intervals, balanced accuracy, MCC, and PR-AUC, provides a more comprehensive characterization of record-level classification performance under class imbalance. Furthermore, the calibration analysis in Table 6 and Figure 9 goes beyond discrimination metrics by assessing the reliability of the predicted probabilities. Collectively, these results provide a more rigorous characterization of classification performance and stochastic variability of the proposed framework.

**Figure 10:**
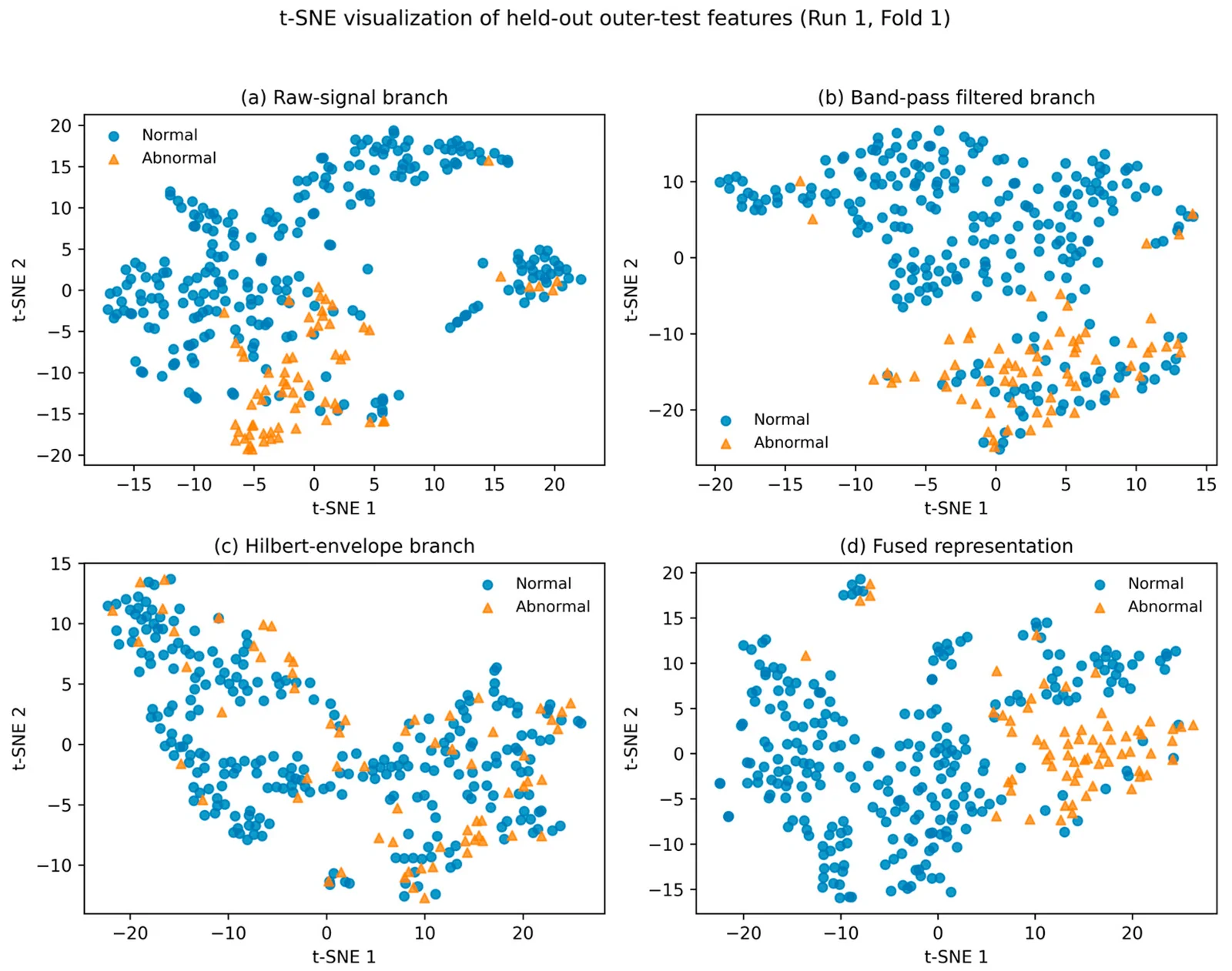
t-SNE visualization of learned representations from held-out outer-test recordings (Run 1, Fold 1): (**a**) raw-signal branch features, (**b**) band-pass filtered-signal branch features, (**c**) Hilbert-envelope branch features, and (**d**) concatenated fused features immediately before the classification head. Circles represent normal recordings and triangles represent abnormal recordings.

To further examine the contribution of multi-branch feature fusion, we applied t-SNE to the learned representations of the held-out outer-test recordings. Figure 10 compares the 128-dimensional representations extracted independently from the raw signal, band-pass filtered, and Hilbert-envelope branches with the 384-dimensional concatenated representation obtained immediately before the classification head. We used the same held-out samples and identical t-SNE settings for all four representations. As illustrated in Figure 10, the individual branches exhibit different class-distribution patterns, supporting their complementary representation characteristics. After feature concatenation, abnormal recordings show a more concentrated organization in part of the fused embedding space, while normal recordings occupy a broader but comparatively distinct region. Although some overlap remains, the fused representation provides a more structured class organization than the individual embeddings. This qualitative analysis supports the role of multi-representation fusion while also indicating that the binary classes are not trivially separable in the learned feature space.

### 4.1. Subject-Disjoint Evaluation and Record-Level Comparison

To investigate the potential influence of subject overlap on reported classification performance, we conducted an additional subject-level sensitivity analysis. The primary experiments in this study used stratified record-level cross-validation on the 3240 PCG recordings. However, reliable subject identifiers were not consistently available for all recordings used in the primary analysis. Therefore, we also performed subject-disjoint evaluation on the training-b subset, for which explicit subject metadata could be reliably identified. This subset contained 490 PCG recordings from 106 subjects, including 386 normal and 104 abnormal recordings. Because subject-independent evaluation is required to assess generalization to unseen individuals, the subject-disjoint experiment is treated as the primary analysis of patient-level generalization for the subset in which reliable subject identifiers are available. The full 3240-record experiment is retained as a record-level benchmark because complete subject identifiers are unavailable for the remaining recordings.

To isolate the effect of the partitioning strategy, we applied two evaluation protocols to the same 490 recordings. First, we performed stratified record-level 10-fold cross-validation, in which different recordings from the same subject could appear in both the training and test partitions. Second, we performed subject-level 10-fold cross-validation by grouping recordings by subject identity, ensuring that recordings from a given subject were assigned exclusively to either the training or test partition within each outer fold. In both experiments, the outer test fold remained completely isolated from model optimization and checkpoint selection. We selected models using an internal validation subset, with balanced accuracy as the checkpoint criterion to reduce bias from class imbalance.

As shown in Table 7, the two partitioning strategies differed substantially. Under record-level cross-validation, TriFusion-PCGNet achieved a mean ROC-AUC of 79.56%, balanced accuracy of 67.91%, and MCC of 0.324. In contrast, subject-disjoint evaluation resulted in a ROC-AUC of 50.31%, a balanced accuracy of 49.20%, and an MCC of −0.017. Sensitivity also decreased substantially from 59.64% to 17.31%, whereas specificity remained comparatively higher.

The marked performance reduction under subject-disjoint evaluation indicates that subject identity and subject-specific acoustic characteristics can substantially influence PCG classification performance. In particular, the decrease in ROC-AUC from 79.56% under record-level splitting to 50.31% under subject-level splitting suggests that record-level evaluation may provide optimistic estimates of generalization when multiple recordings from the same subject can occur across partitions. Nevertheless, this comparison should be interpreted cautiously because it was conducted on only the 490-record training-b subset rather than on the complete 3240-record dataset. Accordingly, for the subset with reliable subject identifiers, the subject-disjoint evaluation is treated as the primary evidence regarding generalization to unseen subjects, whereas the full 3240-record analysis is retained as a record-level benchmark because complete subject identifiers are unavailable for the entire dataset. The substantial performance decrease under subject-disjoint evaluation indicates that the higher record-level results should not be interpreted as evidence of patient-independent generalization. These findings highlight the importance of subject-disjoint partitioning and motivate further validation using larger datasets with complete subject identifiers and independent patient cohorts.

### 4.2. Signal-Duration Sensitivity Analysis

A further signal-duration sensitivity analysis was performed to investigate whether the classification performance was influenced by limiting the duration of each PCG recording to the first 5 s. First, the lengths of all 3240 recordings were examined. Recordings lasted between 5.31 and 122.00 s, with a mean of 22.46 s and a median of 20.83 s. Importantly, no recordings were shorter than 5s. Zero-padding was included in the preprocessing pipeline as a safeguard for shorter signals but was not applied to any recording used in the experiments.

To evaluate the gain of using information beyond the first 5 s segment, the trained outer-fold models were also applied in a full-recording multi-window manner. Each PCG recording was split into consecutive non-overlapping 5 s windows; the model processed each window independently, and the resulting probabilities were averaged to derive a single prediction at the recording level. Hence, compared to the original first-5 s strategy, this analysis used information from the entire available recording. To be consistent with the main evaluation protocol, we kept the same unmodified outer-test partitions and trained models independently.

Most of the classification metrics improved when using the multi-window strategy compared to the first 5 s only (see Table 8). Mean accuracy was increased from 90.87% to 91.49%, sensitivity from 76.94% to 79.10%, F1-score from 77.58% to 79.23%, and balanced accuracy from 85.71% to 86.90%. MCC also increased from 0.719 to 0.739 while precision, specificity and PR-AUC improved less. ROC-AUC slightly decreased from 95.34% to 94.59%. To summarize, these results indicate that using information from the full recording results in a small gain in recording-level classification, especially for the detection of abnormal recordings, while preserving the overall performance characteristics of the proposed model.

To further investigate the effect of the recording length on the performance of the model, we stratified the multi-window results into four duration groups, as shown in Table 9. The best results were achieved for the recordings from 10 to 30 s, with an accuracy of 97.98 ± 0.15%, a balanced accuracy of 95.68 ± 0.64% and an MCC of 0.910 ± 0.007. Recordings of ≥60 s also showed consistently high performance, although this subgroup contained only 41 recordings and should be interpreted with caution. In contrast, the sensitivity was lower (51.34 ± 4.14%) in the 5–10 s group, while the specificity was relatively high (92.13 ± 2.79%). The 30–60 s group showed intermediate performance. These results suggest that classification performance is not entirely invariant with respect to recording duration and that the amount of temporal information contained in a recording can influence model performance. Results are reported, stratified by duration, to give a more transparent characterization of the model’s behavior across recordings of different lengths.

**Table 9:** Duration-stratified performance of the full-recording multi-window evaluation strategy.

Duration	N	Accuracy	Sensitivity	Specificity	Balanced Acc.	MCC
5–10 s	607	83.80 ± 2.72%	51.34 ± 4.14%	92.13 ± 2.79%	71.74 ± 2.99%	0.472 ± 0.080
10–30 s	1780	97.98 ± 0.15%	92.58 ± 1.31%	98.78 ± 0.06%	95.68 ± 0.64%	0.910 ± 0.007
30–60 s	812	82.88 ± 1.21%	79.60 ± 3.86%	84.80 ± 0.52%	82.20 ± 1.76%	0.637 ± 0.030
≥60 s	41	94.31 ± 1.41%	94.87 ± 4.44%	94.05 ± 2.06%	94.46 ± 1.92%	0.873 ± 0.033

### 4.3. Ablation Studies

As shown in Table 10, the extended ablation analysis provides further insight into the contributions of the individual representations and architectural components. The full TriFusion-PCGNet achieved an accuracy of 89.66 ± 0.75%, sensitivity of 73.99 ± 4.49%, balanced accuracy of 83.85 ± 1.69%, and MCC of 0.682 ± 0.020. Among the pairwise branch configurations, Raw + Envelope provided the strongest overall performance, but remained below the full model in sensitivity, F1-score, balanced accuracy, and MCC, supporting the benefit of jointly exploiting all three signal representations.

Component-removal experiments showed that removing attention or BiGRU reduced balanced accuracy from 83.85% to 81.22% and 79.71%, respectively, with corresponding MCC reductions from 0.682 to 0.652 and 0.645. The larger degradation following BiGRU removal particularly supports the contribution of temporal sequence modeling. In contrast, removing dilation slightly increased accuracy (90.00%) and MCC (0.688), indicating that dilation does not provide a consistent independent advantage under this reduced 3-fold ablation protocol. Therefore, its contribution should be interpreted as architecture-dependent rather than as an isolated source of performance improvement.

The additional controls also indicate that the observed performance cannot be explained solely by increased model size or heterogeneous branch backbones. The parameter-matched single CNN achieved a comparable balanced accuracy of 83.17% but a lower sensitivity (72.63%) and F1-score (73.79%) than the full model. Similarly, the single 3-channel CNN achieved high specificity (96.12%) but substantially lower sensitivity (65.26%) and balanced accuracy (80.69%), suggesting that its high overall accuracy was partly driven by the majority class. Mean fusion also underperformed concatenation-based TriFusion-PCGNet in accuracy, sensitivity, F1-score, balanced accuracy, and MCC, supporting the adopted feature-fusion strategy.

From a computational perspective, TriFusion-PCGNet contains 0.688 M trainable parameters and requires approximately 2.003 GFLOPs, with a measured inference time of 5.90 ms per recording. Although the simpler single-stream baselines were computationally faster, the proposed model remains compact in parameter count while providing improved sensitivity and balanced classification performance. These results therefore characterize TriFusion-PCGNet as a trade-off between computational cost and complementary multi-branch representation learning rather than claiming absolute computational superiority.

Computational efficiency was further evaluated using the number of trainable parameters, floating-point operations (FLOPs), GPU memory consumption, and inference time. As reported in Table 10, the complete TriFusion-PCGNet contains approximately 0.688 million trainable parameters and requires 2.003 GFLOPs for processing a 5 s PCG segment. The measured GPU memory consumption was 44.73 MB, while the mean inference time was approximately 5.9 ms per 5 s recording under the evaluated hardware and software configuration. Although these results indicate a relatively compact parameter footprint and short inference latency, the proposed multi-branch architecture is computationally more demanding than several simplified variants. Therefore, we describe the model in terms of its measured performance–complexity trade-off rather than making an unconditional claim of computational efficiency.

These measurements indicate that TriFusion-PCGNet maintains a relatively compact parameter footprint despite its multi-branch architecture. However, several simplified variants exhibit lower FLOPs, memory consumption, or inference latency. Therefore, the computational characteristics of the proposed model are interpreted as a performance–complexity trade-off rather than as evidence of unconditional computational superiority.

## 5. Discussions

To contextualize the performance of TriFusion-PCGNet, Table 11 compares the proposed framework with representative methods previously evaluated on the PhysioNet/CinC 2016 dataset [2,30,39]. Because the evaluation protocols differ across studies, these results are presented for contextual comparison rather than direct ranking.

The 1D-CNN architecture introduced by Xiao et al. [11] prioritizes efficient temporal convolution directly on unprocessed PCG waveforms, highlighting minimal parameter usage. This method exhibits computational efficiency and attains high sensitivity (95.02%), although its specificity (86.01%) reveals challenges in differentiating borderline normal instances, potentially due to dependence on single-domain temporal features.

The HS-Vectors framework [40] presents time-compressed and frequency-expanded embeddings through TDNN architectures and dynamic masking. This approach exhibits enhanced sensitivity (87.61%), underscoring the significance of acquired embeddings; yet it lacks heterogeneous architectural integration.

Ma et al. [19] proposed a parameter-efficient densely connected dual attention network (DDA) to address the problem of PCG classification that is hindered by inherent murmurs and limited supervised heart sound samples. This model operates in a purely end-to-end fashion, taking 1D raw waveform signals as input and hierarchically extracting features from them without any need for manual pre-processing or complex 2D time-frequency transformations. To improve the contextual representation, a parallel dual self-attention mechanism is proposed to explore the semantic interdependencies and long-range correlations in the position and channel dimensions. The signals are processed with a three-second sliding window technique with a one-second stride, and a majority voting decision rule with a 40% anomaly threshold is used for the overall recording-level diagnosis. The DDA model was tested on the challenging PhysioNet/CinC 2016 dataset with stratified 10-fold cross-validation, and the DDA model obtained the state-of-the-art results, with only 0.23 million parameters, an average accuracy of 95.15%, an average overall score (Macc) of 95.24%, an average sensitivity of 97.59%, and an average specificity of 92.89%. Additionally, in its best cross-validation fold (2nd fold), the model obtains a peak accuracy of 97.22% and an overall score of 96.92%, demonstrating an excellent trade-off between diagnostic performance and computational efficiency. In contrast, TriFusion-PCGNet focuses on complementary representation learning by jointly modeling raw, band-pass-filtered, and Hilbert-envelope PCG signals through heterogeneous feature-extraction branches. Importantly, the numerical results are not directly comparable because DDA employs overlapping 3 s signal segmentation, SMOTE-based class balancing, and an empirically selected 40% shard-voting threshold, whereas our evaluation uses an explicitly separated internal validation set for model selection and an untouched outer test fold, repeated across three independent random initializations. Therefore, although DDA reports higher accuracy, the TriFusion-PCGNet results provide a more conservative estimate under the adopted model-selection/evaluation separation while demonstrating the effectiveness of complementary multi-representation feature fusion.

The hybrid 1D CNN + 2D CNN methodology integrates transient waveform examination with spectrogram-centric spatial learning, with an accuracy over 96% [41]. This validates that a multi-space application improves achievement, consistent with the dual-modeling principle embraced in the proposed paradigm.

The Paramps networks [15] use tensor decomposition in convolutional frameworks to diminish redundancy while preserving representational efficacy. Despite attaining an accuracy of 96.40%, Paramps retains an architecturally uniform structure.

Among all the studies analyzed, RAMM + NRBMI + SVM [16] emerges as one of the most robust benchmarks, attaining an accuracy of 98.80% and an F1-score of 99.20%. This approach integrates meticulously produced acoustic characteristics with SVM classification, illustrating that well-designed descriptors can have superior discriminative efficacy. Nonetheless, in contrast to manually created pipelines, the proposed system executes comprehensive end-to-end deep representation learning, obviating the need for manual feature extraction while maintaining an equilibrium between sensitivity and specificity.

DTF-STCANet + KNN framework [29] attains an accuracy of 99.29%, exhibiting balanced sensitivity (99.01%) and specificity (98.94%). The noted enhancement seems to arise from both architectural profundity and organized dual time-frequency amalgamation, diverse backbone incorporation, and geometry-conscious decision-making.

Although the numerical accuracy of TriFusion-PCGNet is lower than several previously reported results in Table 11, these values should not be interpreted as direct evidence of inferior model capability because the underlying evaluation protocols are not identical across studies. The proposed model was evaluated using a strictly separated outer 10-fold cross-validation design in which the outer test fold remained completely untouched during training, epoch selection, and checkpoint selection. In addition, the complete cross-validation procedure was repeated across three independent random initializations, and the reported performance represents the mean across these runs. This protocol provides a more conservative estimate of record-level classification performance than a single split or a single-run cross-validation result. Therefore, the results in Table 11 are presented for contextual comparison rather than direct ranking.

Despite the more conservative evaluation setting, TriFusion-PCGNet maintained an ROC-AUC of 95.34 ± 0.30%, specificity of 94.47 ± 0.31%, and balanced accuracy of 85.71 ± 0.70%. Furthermore, the relatively small variability across independent runs indicates that the model performance is not dependent on a single favorable random initialization.

Several limitations should be acknowledged. The present study addresses binary normal/abnormal PCG recording classification rather than diagnosis of a specific cardiovascular disease. Although calibration was evaluated in the present study, external and prospective validation has not yet been performed. In particular, generalization across independent populations, recording devices, acquisition sites, age groups, and specific cardiac pathologies remains to be established. Future work will therefore focus on subject-level external validation using clinically richer datasets such as CirCor DigiScope and on prospective evaluation under heterogeneous recording conditions. The lower sensitivity than specificity also suggests that the class imbalance may influence abnormal-class recognition. Future work should therefore systematically compare class-weighted BCE, focal loss, and other imbalance-aware optimization strategies under the same evaluation protocol.

## 6. Conclusions

This study presents TriFusion-PCGNet, a hybrid multi-branch deep learning architecture for automated normal/abnormal heart sound classification based on phonocardiogram (PCG) signals. Rather than relying on a single representation, the proposed framework simultaneously processes three complementary deterministic representations of the same PCG signal: the raw waveform, band-pass filtered signal, and Hilbert envelope. Dedicated feature-extraction branches combine residual convolutional learning, dilated convolutions, bidirectional recurrent modeling, and temporal attention. The resulting features are fused into a unified representation for binary normal/abnormal classification.

Extensive experiments on the PhysioNet/CinC 2016 dataset demonstrated stable learning behavior and strong discriminative performance. Under the strictly separated stratified record-level 10-fold cross-validation protocol, TriFusion-PCGNet achieved a mean outer-test accuracy of 90.87%, with mean precision, sensitivity, specificity, F1-score, and AUC values of 78.25%, 76.94%, 94.47%, 77.58%, and 95.34%, respectively. Each outer test fold was kept completely isolated from training and model selection and was evaluated only after checkpoint selection using the corresponding internal validation subset. The results indicate that the model learns discriminative representations for normal/abnormal PCG classification under the evaluated protocol. The extended ablation analysis further demonstrated the respective contributions of the signal representations, temporal modeling, attention, fusion strategy, and model capacity, while the computational analysis characterized the associated performance–complexity trade-off. An additional subject-level sensitivity analysis on the subset with available subject identifiers showed substantially lower performance under subject-disjoint partitioning. Therefore, the primary cross-validation results should be interpreted as estimates of record-level classification performance rather than evidence of patient-independent generalization.

Several limitations should be acknowledged. First, the present study addresses binary normal/abnormal PCG recording classification and should not be interpreted as a diagnosis of a specific cardiovascular disease. Consequently, the ability of the framework to distinguish specific cardiac pathologies has not been established. Second, although probability calibration was assessed in the present study, the model has not yet undergone external or prospective validation. Its generalization across independent populations, recording devices, acquisition sites, age groups, and specific cardiac pathologies therefore remains to be established. Third, although the architecture maintains a relatively compact parameter count, its multi-branch design introduces greater computational cost than simpler single-stream alternatives, which may require further optimization for resource-constrained platforms. Fourth, the adopted 25–400 Hz fourth-order Butterworth filter attenuates out-of-band interference but does not explicitly suppress in-band disturbances such as 50/60 Hz power-line interference; therefore, the robustness of alternative filtering strategies, including adaptive or selectively applied notch filtering, warrants further investigation. In addition, complete subject identifiers were not available for all 3240 recordings used in the primary analysis, preventing subject-disjoint cross-validation on the full dataset. Subject-level evaluation was therefore restricted to the subset with reliable subject metadata. The substantial performance reduction observed under subject-disjoint evaluation highlights the importance of complete subject-level metadata and patient-independent partitioning in future studies.

Future work will extend TriFusion-PCGNet toward clinically richer classification tasks and broader validation settings. In particular, external subject-level validation using datasets such as CirCor DigiScope will be investigated to assess generalization across independent populations, recording conditions, and clinically more detailed murmur labels. Prospective evaluation will also be required to determine performance under real-world acquisition conditions. In addition, pruning, quantization, and knowledge distillation will be investigated to reduce computational requirements, while explainable AI approaches, including attention visualization and feature-attribution methods, will be explored to better characterize the features contributing to model predictions.

Overall, TriFusion-PCGNet demonstrates that integrating complementary representations of the same PCG recording can provide effective automated normal/abnormal heart sound classification. The present findings support further investigation of the framework as a computational approach for automated PCG analysis; however, they do not establish disease-specific diagnostic capability or clinical utility. External and prospective validation across independent clinical populations, devices, and acquisition settings is required before considering clinical application.

## Figures and Tables

**Table 1:** Design rationale of the proposed multi-branch architecture.

Signal Representation	Network Architecture	Extracted Information	Design Rationale
**Raw PCG Signal**	Residual 1D CNN	Morphological characteristics, S1/S2 waveform structures, local acoustic patterns	The raw PCG waveform preserves the complete physiological morphology of heart sounds. Residual learning enables stable optimization of deeper convolutional layers while preserving low-level acoustic information through identity shortcuts.
**Band-pass Filtered PCG Signal**	Dilated Residual 1D CNN	Noise-suppressed temporal patterns, murmur-related frequency components, long-range temporal dependencies	Band-pass filtering removes irrelevant frequency components before feature extraction. Dilated convolutions enlarge the receptive field without increasing the number of trainable parameters, allowing the network to model both local and long-duration pathological events.
**Hilbert Envelope Signal**	Conv1D + BiGRU + Temporal Attention	Energy distribution, cardiac cycle dynamics, systolic/diastolic transitions, temporal importance	The envelope emphasizes the temporal energy profile of heart sounds. BiGRU models bidirectional sequential dependencies, whereas the temporal attention mechanism automatically assigns higher importance to diagnostically informative cardiac events.
**Feature Fusion Layer**	Feature Concatenation	Complementary representation learning	High-level features extracted from the three branches are concatenated to integrate morphological, temporal, and energy-based information into a unified feature representation.
**Classification Head**	Fully Connected Layers + Linear Output; Sigmoid only for inference	Binary classification logit and class probability	The fused feature representation is mapped to a single unactivated logit during training. This logit is passed directly to BCEWithLogitsLoss, which internally combines sigmoid activation with binary cross-entropy. During validation and testing, sigmoid is applied only to convert the logit into a probability, and a threshold of 0.5 is used for the final Normal/Abnormal decision.

**Table 2:** Detailed architectural configuration of TriFusion-PCGNet for reproducibility.

Component	Layer/Operation	Channels/Units	Kernel/Dilation	Stride	Padding	Output/Notes
**Raw branch**	Residual Conv Block 1	1 → 32	k = 15, d = 1	1	7	Two Conv1D + BN + ReLU
	MaxPool1D	—	k = 4	4	0	Temporal downsampling
	Residual Conv Block 2	32 → 64	k = 11, d = 1	1	5	Two Conv1D + BN + ReLU
	MaxPool1D	—	k = 4	4	0	Temporal downsampling
	Residual Conv Block 3	64 → 128	k = 7, d = 1	1	3	Two Conv1D + BN + ReLU
	Global AvgPool1D	—	adaptive	—	—	128-D feature vector
**Filtered branch**	Dilated Residual Block 1	1 → 32	k = 15, d = 1	1	7	Two Conv1D + BN + ReLU
	MaxPool1D	—	k = 4	4	0	Temporal downsampling
	Dilated Residual Block 2	32 → 64	k = 11, d = 2	1	10	Two Conv1D + BN + ReLU
	MaxPool1D	—	k = 4	4	0	Temporal downsampling
	Dilated Residual Block 3	64 → 128	k = 7, d = 4	1	12	Two Conv1D + BN + ReLU
	Global AvgPool1D	—	adaptive	—	—	128-D feature vector
**Envelope branch**	Conv1D	1 → 32	k = 15	1	7	ReLU
	MaxPool1D	—	k = 4	4	0	Sequence length reduced 10,000 → 2500
	BiGRU	32 → 64 per direction	—	—	—	Bidirectional; 128-D sequence features
	Temporal Attention	128 → 1 score	Linear attention	—	—	Softmax over time
	Context vector	—	—	—	—	Weighted sum; 128-D
**Residual projection**	Shortcut Conv1D	in_ch → out_ch	k = 1	1	0	Used only when channel dimensions differ
**Feature fusion**	Concatenation	128 + 128 + 128	—	—	—	384-D fused vector
**Classifier**	Dense	384 → 256	—	—	—	ReLU + BatchNorm
	Dropout	—	*p* = 0.4	—	—	Regularization
	Dense	256 → 64	—	—	—	ReLU
	Dropout	—	*p* = 0.3	—	—	Regularization
	Dense	64 → 1	—	—	—	Linear logit output
**Inference only**	Sigmoid	—	—	—	—	Converts logit to probability; threshold = 0.5

**Table 6:** ECE and Brier Score values for three independent runs.

Run	ECE	Brier Score
Run 1—Seed 42	0.0637	0.0748
Run 2—Seed 123	0.064	0.0776
Run 3—Seed 2024	0.0607	0.07516
Mean ± SD	0.0628 ± 0.0018	0.07585 ± 0.00153
95% CI	0.0584–0.0672	0.07206–0.07964

**Table 7:** Comparison of record-level and subject-level cross-validation on the same 490-record training-b subset.

Metric	Record-Level 10-Fold CV	Subject-Level 10-Fold CV
Accuracy (%)	72.65 ± 6.32	67.55
Precision (%)	42.11 ± 9.52	—
Sensitivity (%)	59.64 ± 18.30	17.31
Specificity (%)	76.18 ± 10.41	81.09
F1-score (%)	47.31 ± 9.96	18.46
ROC-AUC (%)	79.56 ± 5.74	50.31
PR-AUC (%)	61.35 ± 9.19	—
Balanced Accuracy (%)	67.91 ± 6.78	49.2
MCC	0.324 ± 0.113	−0.017

**Table 8:** Performance comparison between the first 5 s and full-recording multi-window evaluation strategies.

Metric	First 5 s	Multi-Window 5 s	Change
Accuracy	90.87 ± 0.17%	91.49 ± 0.65%	0.62
Precision	78.25 ± 0.66%	79.38 ± 1.46%	1.13
Sensitivity	76.94 ± 1.68%	79.10 ± 2.72%	2.16
Specificity	94.47 ± 0.31%	94.69 ± 0.43%	0.22
F1-score	77.58 ± 0.66%	79.23 ± 1.77%	1.65
ROC-AUC	95.34 ± 0.30%	94.59 ± 0.12%	−0.75
PR-AUC	82.43 ± 0.65%	82.75 ± 0.95%	0.32
Balanced Accuracy	85.71 ± 0.70%	86.90 ± 1.38%	1.19
MCC	0.719 ± 0.007	0.739 ± 0.022	0.02

**Table 10:** Ablation analysis for the proposed approach. .

Model/Variant	Accuracy (%)	Sensitivity (%)	Specificity (%)	F1 (%)	Bal. Acc. (%)	MCC	Params (M)	FLOPs (G)	GPU Mem. (MB)	Inference (ms)
**Full TriFusion-PCGNet**	**89.66 ± 0.75**	**73.99 ± 4.49**	93.71 ± 1.54	**74.59 ± 1.72**	**83.85 ± 1.69**	**0.682 ± 0.020**	0.688	2.003	44.73	5.9
Raw + Filtered	89.04 ± 0.69	66.44 ± 14.50	94.87 ± 3.02	70.77 ± 6.23	80.66 ± 5.76	0.653 ± 0.042	0.656	1.8	36.99	1.89
**Raw + Envelope**	89.35 ± 0.91	69.32 ± 4.82	94.52 ± 1.82	72.76 ± 1.91	81.92 ± 1.78	0.665 ± 0.025	0.656	1.103	64.87	4.06
Filtered + Envelope	87.07 ± 0.56	69.17 ± 1.21	91.69 ± 0.40	68.71 ± 1.36	80.43 ± 0.81	0.606 ± 0.017	0.656	1.103	62.24	3.68
Full − Dilation	**90.00 ± 1.30**	72.18 ± 4.83	94.60 ± 2.10	**74.78 ± 2.73**	**83.39 ± 1.92**	**0.688 ± 0.036**	0.688	2.003	64.9	5.61
Full − Attention	88.95 ± 1.10	68.12 ± 4.00	94.33 ± 2.36	71.71 ± 1.06	81.22 ± 0.91	0.652 ± 0.020	0.688	2.003	68.6	5.6
Full − BiGRU	88.95 ± 0.54	64.05 ± 7.71	95.38 ± 2.41	70.29 ± 2.26	79.71 ± 2.70	0.645 ± 0.013	0.655	1.831	39.52	2.55
Same CNN ×3	89.75 ± 1.17	68.86 ± 6.74	95.14 ± 2.63	73.36 ± 2.40	82.00 ± 2.35	0.676 ± 0.028	0.918	2.7	39.9	2.67
3-channel Single CNN	89.78 ± 0.97	65.26 ± 4.15	**96.12 ± 2.10**	72.41 ± 1.37	80.69 ± 1.20	0.671 ± 0.024	0.318	0.921	42.62	1.16
Parameter-matched Single CNN	89.38 ± 1.24	72.63 ± 1.19	93.71 ± 1.82	73.79 ± 1.96	83.17 ± 0.49	0.672 ± 0.028	0.668	2.041	**35.03**	**0.92**
Mean Fusion TriFusion	88.46 ± 2.47	70.22 ± 4.39	93.16 ± 4.23	71.64 ± 3.27	81.69 ± 0.13	0.649 ± 0.058	0.623	2.003	67.92	5.42

Bold values represent the best performance metrics.

**Table 11:** Performance results of existing approaches evaluated on the 2016 PhysioNet/CinC.

Methods	Evaluation Protocol	Accuracy (%)	Specificity (%)	F1-Score (%)	Sensitivity (%)
HS Model [40]	10-fold CV	95.6	97.7	89.2	87.6
1D + 2D CNN [41]	10-fold CV	96.3	97.9	91.1	90.6
CNN [11]	10-fold CV (only training dataset)	93.0	86.0	91.0	95.0
ParaCNN Model [15]	10-fold CV	94.6	94.2	86.3	85.1
ParaMPS [15]	10-fold CV	96.1	98.7	88.7	86.0
Params [15]	10-fold CV	96.4	99.1	89.3	86.5
DDA model [19]	Stratified 10-fold CV	95.1	92.9	-	97.6
TriFusion-PCGNet	Strictly separated stratified 10-fold outer CV, 3 independent runs	90.87 ± 0.17	94.47 ± 0.31	77.58 ± 0.66	76.94 ± 1.68

## Data Availability

The original data presented in the study are openly available at https://www.kaggle.com/datasets/bjoernjostein/physionet-challenge-2016 (accessed on 18 August 2026).

## Supplementary Materials

The following supporting information can be downloaded at: https://www.mdpi.com/article/10.3390/life16091417/s1.

## Appendix A

**Table A1:** Dataset composition and provenance of the 3240 PCG recordings included in the analysis. Explicit subject identifiers were available for 490 recordings from 106 unique subjects; subject identifiers for the remaining recordings were not inferred.

Source Subset	Normal	Abnormal	Total	Subject ID Available
training-a	117	292	409	No
training-b	386	104	490	Yes
training-c	7	24	31	No
training-d	27	28	55	No
training-e	1958	183	2141	No
training-f	80	34	114	No
Total	2575	665	3240	490 recordings

**Table A2:** Summary of duplicate auditing and subject-level metadata. All 301 recordings in the separate validation directory had exact counterparts in the training data according to both recording identifiers and SHA-256 hashes and were therefore excluded from the analytical dataset. Subject-disjoint evaluation was performed only on recordings with explicit subject identifiers.

Item	Result
Training recordings included in analysis	3240
Separate validation recordings inspected	301
Validation recordings matching training by recording ID	301/301 (100%)
Validation recordings matching training by SHA-256 hash	301/301 (100%)
Validation recordings included in analysis	0
Recordings with explicit subject IDs	490
Unique subjects	106
Subject IDs inferred from filenames	No
Subject-disjoint evaluation	10-fold StratifiedGroupKFold

The complete recording-level manifest is provided in Supplementary Table S1, the detailed duplicate-audit results in the Supplementary Duplicate Check Report, and the available subject-to-recording mapping together with the corresponding subject-disjoint fold assignments in Supplementary Table S2.
